# The diverse phenotypic and mutational landscape induced by fluoroquinolone treatment

**DOI:** 10.1128/msystems.00713-25

**Published:** 2025-07-31

**Authors:** Sayed Golam Mohiuddin, Pouria Kavousi, Diego Figueroa, Sreyashi Ghosh, Mehmet A. Orman

**Affiliations:** 1 William A. Brookshire Department of Chemical and Biomolecular Engineering, University of Houston 14743, Houston, Texas, USA; Iowa State University 1177, Ames, Iowa, USA

**Keywords:** antibiotic resistance, antibiotic tolerance, resistant mutants, adaptive laboratory evolution, fluoroquinolone, mutagenesis, bacterial fitness factors

## Abstract

Antibiotic resistance remains a major public health challenge, yet the broader effects of antibiotic treatment on bacterial tolerance, resistance, and fitness are not fully understood. In this study, we investigated how *Escherichia coli* adapts to fluoroquinolone stress using adaptive laboratory evolution. Cell populations were subjected to repeated cycles of high-dose ofloxacin exposure followed by drug-free recovery, a dynamic model that imposes alternating selective pressure and inter-strain competition, reflecting real-world clinical or environmental conditions. Our results demonstrate that tolerance and resistance can evolve independently, even under identical conditions, leading to diverse phenotypic outcomes. Notably, we observed the emergence of mutants with high ofloxacin tolerance but reduced minimum inhibitory concentrations, an outcome that challenges conventional understanding. Fitness traits, including lag phase duration, doubling time, competition score, redox activity, and ATP levels, were variably affected across evolved strains, with no consistent correlation between fitness and tolerance or resistance. Whole-genome sequencing revealed both known and novel mutations, with limited convergence across populations. For example, while mutations in the *icd* gene were commonly observed, many other mutations, including in *cyoE, lgoT, yghC*, *rnd*, *dld*, and *uidB*, were unique to individual lineages. The lack of convergence across evolved populations may reflect the influence of competitive dynamics during recovery phases, where differing growth advantages shape selection in parallel with antibiotic pressure. These findings underscore the complexity of microbial adaptation and highlight how fluctuating environments and population-level interactions can drive non-uniform evolutionary outcomes.

**IMPORTANCE:**

Antibiotic resistance poses a critical global health threat, with antibiotic-tolerant cells further complicating treatment by promoting infection relapse and enabling resistance mutations. Though tolerant cells can evolve into resistant strains, their phenotypic and genotypic characteristics are still poorly understood. In this study, we used adaptive laboratory evolution to generate several distinct ofloxacin-resistant mutants and examined their fitness (e.g., lag phase), metabolic traits (e.g., ATP levels), and genetic adaptations through whole-genome sequencing. We uncovered novel findings, including highly tolerant mutants exhibiting unexpectedly low minimum inhibitory concentrations and others with shorter lag phases, challenging conventional patterns in bacterial resistance evolution. Our findings provide critical insights into the diverse pathways and mechanisms underpinning bacterial adaptation, underscoring the complexity of resistance evolution.

## INTRODUCTION

Referred to as the hidden pandemic (1), the evolution of resistant mutations that render antibiotic therapy ineffective has been identified as a serious global public health concern (2, 3). According to a United Nations Environment Program report, antibiotic resistance will kill about 10 million people by 2050, which will potentially match the number of fatalities from cancer (4, 5). A reduction in treatment options could push public health into a post-antibiotic era that can significantly increase the number of infectious disease cases (6). Numerous mechanisms, including reversible and irreversible processes that bacteria can use to survive antibiotics, have been linked to the failure of antibiotic therapies. Bacterial persistence is a reversible survival mechanism that allows a small fraction of a genetically identical cell population to tolerate high doses of antibiotics transiently (7–11). This is distinct from antibiotic resistance, which is characterized by a heritable increase in the minimum inhibitory concentration (MIC) of antibiotics, typically arising through genetic mutations (12–15). While resistance and persistence are two different phenomena, they might be related, given that persister cells can act as a reservoir for the formation of resistant mutations (16–22). Additionally, cells may evolve mechanisms that enhance survival during antibiotic exposure without increasing MIC. To distinguish this phenomenon from classical resistance, we refer to it as “tolerance” in this study.

Bacteria acquire antibiotic resistance not only through chromosomal mutations but also via horizontal gene transfer (HGT), which enables the exchange of genetic material through mechanisms such as conjugation, transformation, and transduction. HGT facilitates the rapid spread of resistance genes across species and environments, contributing significantly to the global rise in antimicrobial resistance (23–25). Furthermore, a variety of environmental factors, such as nutrient depletion, UV radiation, antibiotics, and toxic chemicals, can alter the genetic makeup of bacterial chromosomes and may lead to structural anomalies, deletions, insertions, and single nucleotide polymorphisms that can make the cells tolerant and/or resistant (16, 17, 26–29). In particular, the extensive use of antibiotics that damage DNA, like fluoroquinolones, can significantly increase bacterial mutagenesis by activating error-prone DNA repair systems (17, 30–36). Mutations that result in antibiotic resistance may modify drug target sites, efflux pumps, membrane structure, metabolic pathways that may enhance cell dormancy, and enzymes that deactivate antibiotics (37–40). Although antibiotic resistance has been extensively studied (41–44), the potential impact of antibiotic treatment on bacterial resistance and persistence is a matter of great concern, underscoring the need for further research in this area.

Adaptive laboratory evolutionary (ALE) experiments provide critical insights into the mechanisms by which bacteria adapt and develop resistance in response to antibiotic exposure (16, 17, 19, 21, 45). For example, Nathalie Balaban and her research group (16) demonstrated through *in vitro* evolution experiments with ampicillin treatment that tolerance facilitates the emergence of antibiotic-resistant mutants. Experimental data in combination with the hypothetical model demonstrated that persistence is pleiotropically linked to increased mutation rates and consequently acts as the source of antibiotic-resistant mutants. Using classical and metabolism-dependent antibiotic-mediated evolutionary experiments, James Collins (21) and his research group demonstrated the presence of mutations in the metabolic genes that were shown to be clinically relevant. Campey et al. (46) found that bacteria in well-mixed environments developed common resistance mutations (e.g., *gyrA* S83L), whereas those in structured environments exhibited a broader range of adaptations, including mutations in efflux pump regulators and morphological changes such as cell shrinkage during division (46). Ching and Zaman (47) showed that even low doses of ciprofloxacin over time can lead to strong, stable multi-drug resistance through both known and new mutations (47). Huseby et al. (48) also found that different bacterial populations exposed to the same treatment evolved resistance differently, both in terms of genetics and behavior.

ALE experiments, persistence, and mutagenesis are intricately linked to bacterial adaptation and antibiotic resistance. During ALE experiments, persister cells, variants that survive initial treatments, play a crucial role as a reservoir for survival (16, 17, 19, 21). Although not inherently resistant, these cells can undergo mutagenesis, especially when exposed to stressors like antibiotics that induce DNA damage and error-prone repair mechanisms (17, 19, 21, 49). The cyclic nature of ALE, involving alternating phases of antibiotic exposure and recovery, further amplifies this process by allowing surviving cells to proliferate and compete, potentially acquiring and fixing beneficial mutations that confer resistance. Unlike traditional models, which suggest that resistance mutations typically come with fitness costs such as slower growth rates (16, 50–53), the dynamic environment of cyclic ALE can favor the selection of mutations that confer resistance while maintaining overall fitness. These adaptive responses to fluctuating environments, commonly observed in real-life clinical and environmental settings (54, 55), underscore the need for further studies to clarify how these processes impact bacterial fitness.

Although the convergence of evolutionary trajectories toward a common pathway or mechanism is highly sought after by the scientific community (16, 17, 19, 21, 56), a crucial aspect for identifying therapeutic targets, living organisms have continuously evolved, resulting in complex genetic landscapes. This diversity has given them a multitude of mechanisms to survive adverse conditions. To address critical gaps in our understanding of antibiotic resistance evolution, this study combines ALE, phenotypic characterization, whole-genome sequencing, and gene knockout experiments to explore the complex interplay between tolerance, resistance, and fitness in *Escherichia coli*. By systematically assessing fitness-related parameters, including lag time, doubling time, competition score, redox activity, and ATP levels, we found no consistent trade-off between fitness and antibiotic adaptation in evolved populations subjected to intermittent ofloxacin exposure. This ALE model, which mimics real-world fluctuations in antibiotic pressure and allows for natural competition, revealed substantial phenotypic and genetic diversity. Notably, we identified highly tolerant strains with low MICs, a phenotype that departs from classical definitions of resistance. Whole-genome sequencing, along with functional validation and targeted gene knockouts, revealed both known and novel genetic determinants of antibiotic tolerance and resistance. Collectively, these findings provide new insights into the complexity and unpredictability of bacterial adaptation under antibiotic stress and contribute to a deeper understanding of resistance evolution.

## RESULTS

### Adaptive laboratory evolutionary experiments generated diverse tolerant and resistant strains

**Fig 1:**
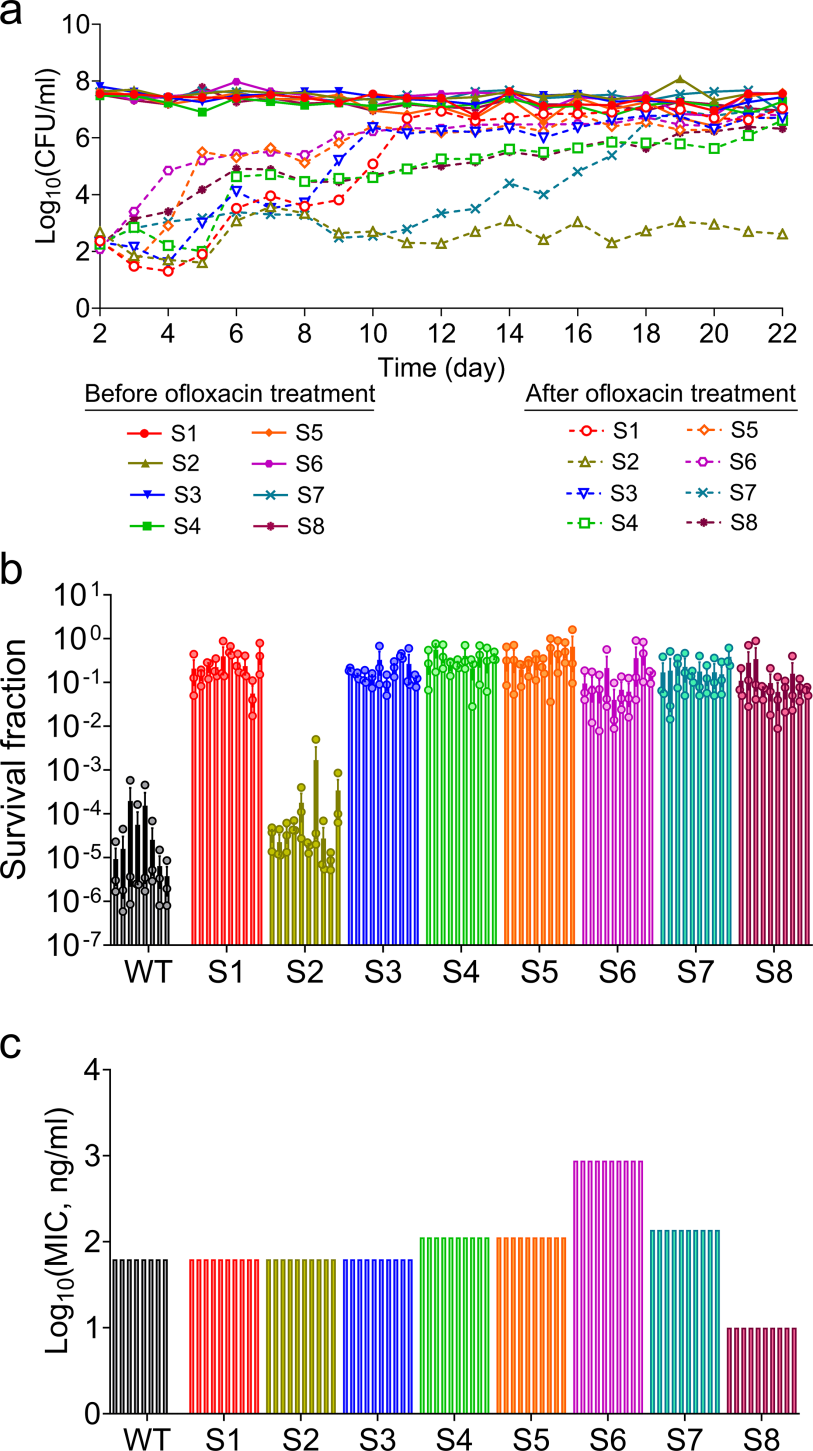
Generation of ofloxacin mutant strains using adaptive laboratory evolutionary experiment. (**a**) Stationary phase *E. coli* MG1655 MO cells (16-h culture) carrying the pUA66-empty vector were diluted 1:100 in LB and treated with ofloxacin (5 µg/mL) for 7 h. After washing, half of the cells were transferred to fresh LB for overnight recovery, while the other half were plated to determine colony-forming units (CFU). This cycle was repeated daily for 22 days. CFU levels before and after treatment are shown in the plot. *n* = 1. (**b**) Stationary phase cells of 10 randomly picked colonies of the indicated strains were diluted 100-fold in LB and treated with ofloxacin (5 µg/mL) for 7 h. After the treatment, cells were washed and plated on LB agar to determine the survival fractions. *n* = 10 (10 independent colonies; each tested in triplicate). (**c**) MICs of 10 randomly selected colonies from each sample (88 total, including wild type) were measured using the twofold microdilution method in 96-well plates, with antibiotic concentrations adjusted to match the ETEST conditions (note: MICs for samples S1–S8 were determined using ofloxacin ETEST strips; see Fig. S2). MICs were calculated as the average of the lowest concentration showing visible growth and the highest showing no growth. *n* = 10 (10 independent colonies). Data corresponding to each time point represent mean value ± standard error.

We used an *in vitro* ALE methodology to investigate whether intermittent antibiotic exposure could yield mutants displaying increased tolerance or resistance. To carry out this experiment, we utilized an *Escherichia coli* MG1655 strain (MO-cured [57]) harboring both a chromosomally integrated inducible mCherry expression system and a pUA66-empty vector (EV) with kanamycin resistance gene (Table S1). Kanamycin (50 µg/mL) was used to maintain the plasmid and prevent contamination, given the prolonged duration of the experiment. We used mCherry-expressing cells, which proved highly useful in analyzing the fitness characteristics of the generated mutant strains. We initiated the ALE experiment by preparing eight separate 2 mL overnight cultures from the same cell stock and incubating them under identical conditions in a shaker at 37°C and 250 rpm for 16 h. These cultures were then diluted in 2 mL of fresh lysogeny broth (LB) medium and promptly exposed to a high concentration of ofloxacin (5 µg/mL, 100× MIC) for 7 h (Fig. S1). This concentration was selected based on its clinical relevance (fluoroquinolones can reach similar levels in patient tissues) (48, 58, 59) and its widespread use in experimental settings to enrich persister cells. The 7-h exposure duration follows established protocols, effectively eliminating antibiotic-sensitive cells while enriching for persisters, as evidenced by the characteristic biphasic killing pattern (Fig. S1) (7, 8). After the 7-h treatment, cells were collected and washed with phosphate-buffered saline (PBS) to remove antibiotics, and half of the washed cells were plated to quantify the surviving cells. The remaining cells were inoculated in 2 mL of fresh LB medium and allowed to grow overnight (16 h) with shaking at 37°C to facilitate the recovery of the surviving cells, and this cycle was repeated over a span of 22 days. To visualize the trajectory of survival, cells were plated on LB agar plates both before and after the ofloxacin treatment in each cycle of the ALE experiment (Fig. 1a). Throughout the course of the experiment, the trajectories of eight distinct samples exhibited varying forms of adaptation to the high concentration of ofloxacin. Sample 2 (S2) displayed no discernible increased survival to ofloxacin even after intermittent exposure for 22 days (Fig. 1a), akin to the parental strain (without 22 days of ALE). Conversely, seven samples (S1, S3, S4, S5, S6, S7, and S8) exhibited an increased survival to ofloxacin (Fig. 1a) compared to the control (parental strain, WT).

Given the random nature of mutations, each of the eight samples may harbor distinct and numerous ofloxacin mutants. To determine whether these mutants display consistent phenotypic traits within each sample, we streaked cells from each sample onto LB agar plates. We then randomly selected at least 10 individual colonies from each sample to assess their tolerance and/or resistance properties by measuring ofloxacin survival fractions and MIC levels. Although there is noteworthy variation in survival fractions among the eight samples, as depicted in Fig. 1a, the survival fractions among the 10 colonies within each sample were found to be similar in Fig. 1b. Our findings also revealed consistent MIC levels among the 10 selected colonies within each sample, and most strains displayed MIC levels close to that of the parental strain (Fig. 1c). Interestingly, the MIC level of sample S8 was significantly lower than that of the parental strain despite its markedly higher survival fractions (Fig. 1b and c). This trend differs in sample S6, as we observed both higher MIC levels and increased survival to ofloxacin compared to the parental strain (Fig. 1b and c). When we performed a correlation analysis across all samples (Fig. S2), we observed no strong relationship between survival fractions and MIC levels (*R*^2^ = 0.004, Pearson correlation). Altogether, these results highlight two key findings: (i) mutants within each sample display highly consistent survival and MIC values, despite variability across samples. (ii) High survival can occur independently of MIC levels, with some mutants showing high tolerance despite low resistance (i.e., low MIC).

Collateral effects of antibiotic resistance occur when resistance to one antimicrobial alters susceptibility to another, leading to either collateral resistance or collateral sensitivity (60–62). In our ALE experiments with intermittent ofloxacin exposure, we used a plasmid encoding kanamycin resistance to prevent contamination. Since kanamycin could potentially introduce collateral effects, we conducted a parallel 22-day ALE experiment with the parental MG1655 strain in the absence of kanamycin selection. By day 10, several cultures had developed significantly increased ofloxacin tolerance relative to day 1, closely resembling the tolerance patterns observed in the kanamycin-treated ALE experiments (Fig. 1a; Fig. S3a). By day 22, most evolved strains had acquired notable ofloxacin resistance, again mirroring outcomes seen with kanamycin (Fig. 1a; Fig. S3a). Interestingly, one population remained similar to the day-1 phenotype until day 18, after which its tolerance increased 10-fold by day 20 and again by day 22 (Fig. S3a). MIC measurements showed that six populations had lower MICs than the parental strain, while three exhibited higher MICs (Fig. S3b). Although some variability is expected due to the stochastic nature of evolution, these results are consistent with the two key observations from the original ALE experiments: the emergence of distinct phenotypic traits across independently evolved populations despite identical conditions, and the lack of correlation between tolerance and resistance (specifically, the emergence of strains with high tolerance and reduced MICs). Overall, the similarity in outcomes suggests that the presence of kanamycin may not have had a substantial impact on evolutionary dynamics.

### Antibiotic-tolerant cells exhibit diverse fitness attributes

A trade-off can exist between antibiotic tolerance/resistance and other traits. For example, mutations that confer antibiotic resistance may come with associated costs, potentially reducing the bacterium’s overall fitness. To explore the connections between the tolerance/resistance of our samples and their fitness, we evaluated various fitness indicators, such as the lag score, doubling time, competition score, non-growing score, cellular redox activities, and ATP levels.

**Fig 2:**
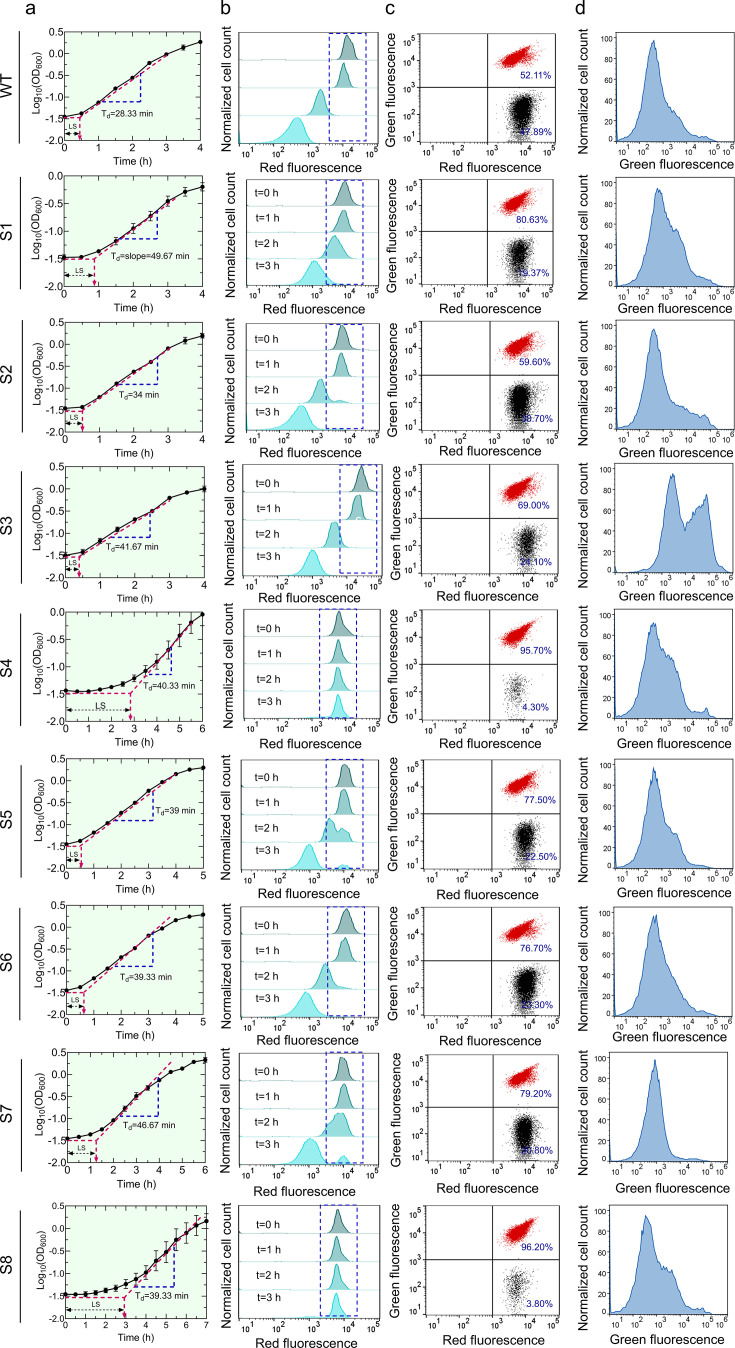
Determination of fitness factors and metabolic activities of the evolved strains. (**a**) Growth curves of the mutants and the WT strain (control). Stationary phase cells were diluted 100-fold in LB media and cultured for 24 h. At designated time points, cells were collected to measure OD_600_ using a plate reader. These growth curves were utilized to determine the lag scores and doubling times (for details, see Materials and Methods). *n* = 4. (**b**) Flow-cytometry-based approach for the quantification of non-growing cell levels. The mutants and WT cells (harboring an isopropyl β-D-1-thiogalactopyranoside [IPTG]-inducible mCherry expression cassette in their genome) were cultured overnight in the presence of 1 mM IPTG to express the mCherry protein. Stationary phase cells were collected and washed to remove the IPTG and diluted 100-fold in LB media and grown in a shaker in the absence of IPTG. Cell division along with protein dilution was monitored using a flow cytometer at single-cell levels at the indicated time points. Non-growing cells retained their mCherry levels. A representative biological replicate is shown, with all three biological replicates consistently yielding similar trends. *n* = 3. (**c**) Competition assays for mutants and WT strains in cocultures. Mutants (harboring pUA66-EV) and WT cells (harboring pUA66-*gfp*) were cultured individually overnight (16 h) in the presence of 1 mM IPTG in LB media. Stationary phase cells of the mutants and WT cells were diluted 100-fold in LB and cocultured in the presence of 1 mM IPTG for 24 h. At *t* = 24, cells were collected, diluted in PBS, and analyzed with a flow cytometer at the single-cell level, showing two distinct cell populations. For WT, red dots represent WT cells carrying both mCherry and green fluorescent protein (GFP) expression systems, while black dots represent WT cells carrying only the mCherry expression system. For samples S1–S8, red dots represent WT cells carrying both mCherry and GFP expression systems, while black dots represent the mutant cells carrying only the mCherry expression system. A representative biological replicate is shown, with all three biological replicates consistently yielding similar trends. *n* = 3. (**d**) Redox Sensor Green (RSG) staining of stationary phase cells. Stationary phase mutants and WT cells were stained with RSG dye and analyzed with a flow cytometer to measure their metabolic activities. A representative flow cytometry diagram is shown. All independent biological replicates showed a similar trend. *n*  =  3. Data corresponding to each time point represent the mean value ± standard error.

**Fig 3:**
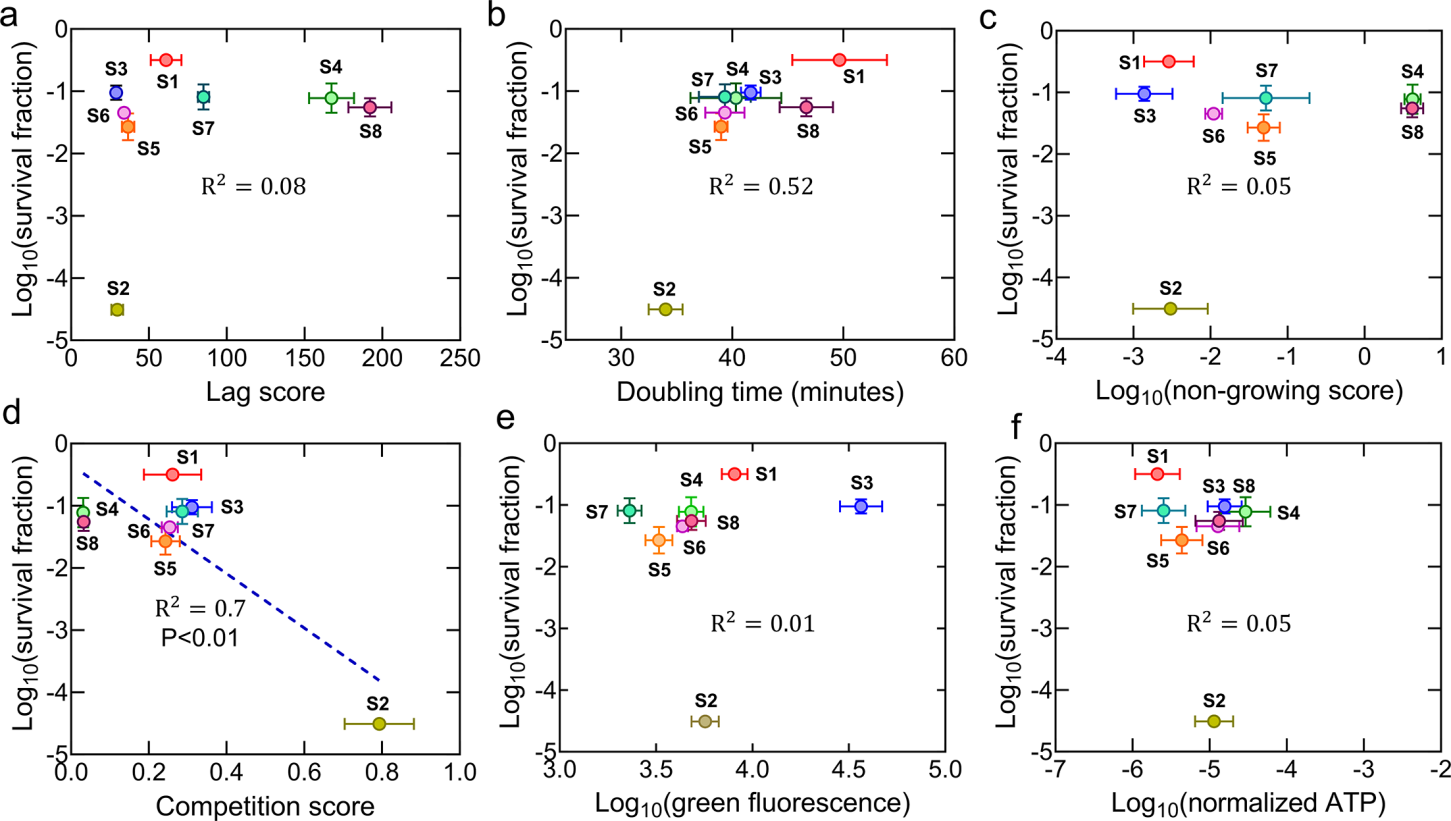
Correlation between survival fractions, fitness factors, and metabolic parameters of mutant strains. All data for this analysis are from Fig. 2a through d and Fig. S4a through f. Correlation between survival fractions and (**a**) lag scores, (**b**) doubling times, (**c**) non-growing scores, (**d**) competition scores, (**e**) cellular redox state, and (**f**) ATP levels. Pearson correlation was performed between survival fractions and each parameter for the mutant and WT strains. *P* values (for statistically significant correlations) and *R*^2^ values are shown on the graphs. Data corresponding to each time point represent the mean value ± standard error.

The lag score, representing the duration that bacteria take to initiate growth in a specific medium, was determined using the methodology outlined in previous studies (63, 64). It was evident that samples S4 and S8 exhibited a notable increase in lag scores compared to the control (Fig. 2a; Fig. S4a). Conversely, samples S2, S3, S5, and S6 displayed lag scores like the control (Fig. 2a; Fig. S4a). Our attempt to correlate survival fraction data with the lag score of each sample was motivated by existing literature, which suggests that cell tolerance to antibiotics is influenced by the length of the lag phase, where a longer lag phase corresponds to higher antibiotic tolerance (16, 50). Our findings revealed no clear correlation between the lag scores and survival fractions of all eight samples (*R*^2^ = 0.08, Pearson correlation, Fig. 3a). Another parameter potentially contributing to the higher survival fraction of the ALE-evolved strains is doubling time. Apart from sample S2, all other evolved samples exhibited higher doubling times compared to the control strain (Fig. 2a; Fig. S4b). Unlike lag scores, when comparing survival fractions and doubling times for all samples, we found a moderate correlation between these two parameters (*R*^2^ = 0.52, Pearson correlation, Fig. 3b), indicating that doubling time may be associated with enhanced tolerance or resistance, although this relationship did not reach statistical significance.

The non-growing score, another significant parameter in defining fitness, was determined as the fraction of cells that did not undergo the cell division process at a specific time compared to the levels of actively growing cells. The non-growing population is heterogeneous and includes antibiotic-sensitive cells, persisters, and “viable but non-culturable” cells (65–67). Although persisters may constitute only a small fraction (~4%) of the non-growing cell population (67), this metric provides insight into the physiological state of the population under stress and helps distinguish between cells that are proliferating and those in a dormant or inactive state. To determine the non-growing cell levels, we employed a fluorescent protein-dilution technique that we commonly utilized for monitoring cell growth (57, 65, 68). In this process, we induced mCherry expression during the overnight growth of an *E. coli* strain containing a chromosomally integrated isopropyl β-D-1-thiogalactopyranoside (IPTG)-inducible mCherry expression cassette (57, 65). The mCherry-positive cells from overnight cultures were then diluted in fresh medium and cultured without the inducer. Initially, all cells exhibited high levels of mCherry protein, which gradually decreased with cellular growth due to protein dilution. However, a specific subpopulation still maintained elevated levels of red fluorescence owing to their lack of division. We assessed non-growing cell levels in each evolved sample at 3 h of growth (a mid-exponential point used to distinguish growing and non-growing subpopulations in the flow diagram [65–67]) and found that S4 and S8 exhibited significantly delayed growth resumption compared to the control strain (Fig. 2b; Fig. S4c), which is expected as they had higher lag scores (Fig. 2a). In contrast, samples S1, S2, S3, S5, and S6 showed little or no non-growing cells, similar to the parental strain, while S7 had significantly more non-growing cells than the parental strain (Fig. 2b; Fig. S4c). No definitive correlation emerged when we examined the correlation between non-growing scores and survival fractions (*R*^2^ = 0.05, Pearson correlation, Fig. 3c).

Competition scores represent the ratio of the mutant strain to the parental strain at a specific growth phase when they are cultured together. Our evolved strains contain an IPTG-inducible mCherry protein expression cassette in their genomic DNA and an empty plasmid (pUA66-EV). The parental strain, which also carries the mCherry expression system, contains pUA66-*gfp* plasmids encoding the green fluorescent protein (GFP). Cocultures were prepared by diluting and combining both the evolved and parental cells from separate overnight cultures into 2 mL fresh LB media in equal numbers (determined by a flow cytometer). Following 24 h of culturing, the cocultures were analyzed using a flow cytometer to quantify mutant cells (mCherry only) and parental cells (mCherry + GFP), enabling the calculation of competition scores (Fig. 2c; Fig. S5 for time-dependent flow cytometry data). We also utilized a parental strain with the empty plasmid as a control (black dots in the flow diagram for WT in Fig. 2c) to assess the impact of GFP overexpression on the competition score. While almost all mutant strains exhibited slower growth in co-cultures compared to the parental strain, S4 and S8 displayed significant growth inhibition, suggesting their inability to compete with parental cells (Fig. 2c; Fig. S4d). Upon comparing survival fractions and competition scores for all samples, we found a moderate correlation between these two parameters (*R*^2^ = 0.7, Pearson correlation, *P* < 0.01, Fig. 3d), indicating that the ability of a strain to survive and its competitive advantage in coculture settings may be interconnected. This is consistent with the correlation analysis of doubling time scores (Fig. 3b), as fast-growing strains are expected to compete better.

We evaluated the metabolic activities of mutant strains with Redox Sensor Green (RSG) dye and intracellular ATP measurements. RSG dye, capable of entering live bacteria, emits a green fluorescence signal when it undergoes reduction by bacterial reductase enzymes. RSG and ATP measurements were conducted immediately after diluting the overnight cultures, consistent with the ofloxacin treatment conditions. Interestingly, while the redox activities of most mutant strains were very similar to those of the parental cells, a significant increase in the redox activity of S3 was observed compared to that of the parental strain (Fig. 2d; Fig. S4c). On the other hand, the S4 strain exhibited slightly higher ATP levels, while S1, S5, and S7 demonstrated considerably lower ATP levels compared to the parental strain, though these differences were not statistically significant (Fig. S4f). We did not observe a correlation between cell survival fractions and RSG or ATP levels (Fig. 3e and f). This lack of correlation may stem from the multifaceted nature of bacterial survival strategies, which should involve various cellular processes beyond metabolic activity.

### Whole-genome sequencing revealed diverse genetic attributes

**Fig 4:**
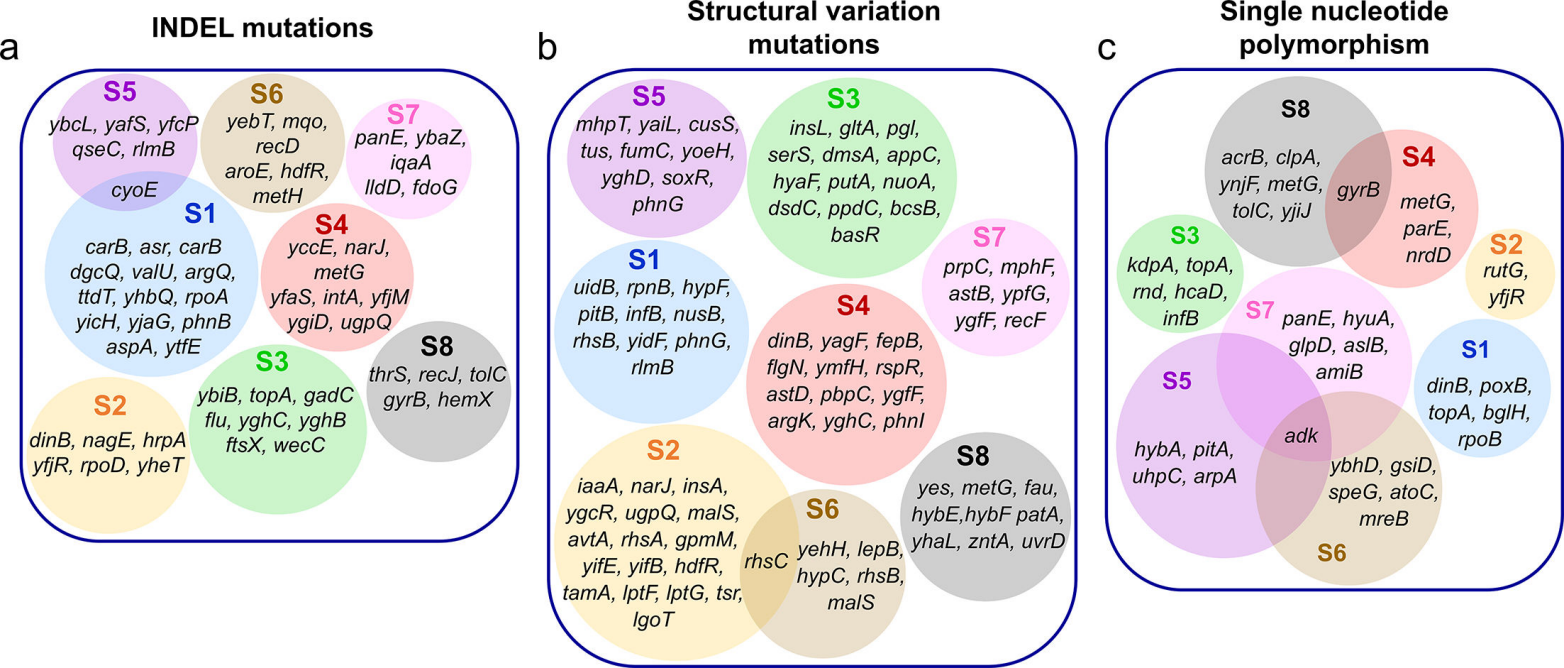
The whole-genome sequencing reveals that various types of mutations have occurred within the evolved strains. A Venn diagram illustrates the mutations identified in the mutants as a result of the adaptive laboratory evolution experiment, including (**a**) insertions and deletions, (**b**) structural variations, and (**c**) single nucleotide polymorphisms.

In a clonal population, the development of high antibiotic tolerance and resistance should be attributed to the accumulation of mutations; therefore, we conducted comprehensive whole-genome sequencing for each sample (see Materials and Methods). The sequencing data revealed three distinct types of mutations: insertions and deletions (INDELs), structural variations (SVs), and single nucleotide polymorphisms (SNPs) (Table S2). Although mutations in the same genes were occasionally observed across the samples, there was no evidence that the evolution of resistant mutants in these samples converged on a common pathway or mechanism (Fig. 4). The Venn diagram illustrates the distribution of mutations within the samples, highlighting that only a select few genes, such as *cyoE* (present in S1 and S5), *rhsC* (present in S2 and S6), *gyrB* (present in S4 and S8), and *adk* (present in S5, S6, and S7), exhibited these common mutations within their genomes (Fig. 4). However, we noted SNP mutations in one of the metabolic genes, *icd*, which were present in most samples except S4 and S6 (Table S2), aligning with existing literature (21). To understand how mutations identified in the evolved strains might influence antibiotic resistance, tolerance, and fitness, we selected 50 genes (prioritizing those most frequently mutated across evolved samples) for further investigation. These genes represent a range of functional categories, including energy metabolism, transport, DNA repair, regulation, and cell envelope processes, as well as genes with unknown roles (Table 1). This functional diversity reflects the broad cellular responses associated with antibiotic adaptation. Although introducing the exact mutations (SNPs, INDELs, and SVs) would provide the most precise mechanistic insights, replicating these mutations experimentally is technically challenging, particularly given their complexity and the presence of multiple mutations per strain. To address this, we first applied *in silico* tools to predict the likely impact of these mutations (see Materials and Methods for details). The selected genes predominantly contain INDELs and SVs, and *in silico* translation of their mutated sequences frequently revealed premature stop codons or disrupted stop signals (Table 1), potentially resulting in loss of function (69, 70). For SNPs, we used PolyPhen-2 to predict the effects of amino acid substitutions (71), with most mutations classified as likely damaging (Table 1).

**TABLE 1:** Protein functionality prediction after DNA mutations

Mutant	Gene function^*a*^	Mutation type	Functionality after mutation
Δ*icd*	TCA enzyme; aids metabolism and stress response	SNP	Damaging (PolyPhen-2)^*b*^
Δ*cyoE*	CyoE makes heme O for cytochrome bo	IN	Premature termination^*c*^
Δ*lgoT*	Proton-driven L-galactonate uptake transporter	DEL	Premature termination
Δ*yghC*	Uncharacterized glycolate operon protein	IN	Premature termination
Δ*tolC*	TolC is an outer membrane efflux channel	IN	Premature termination
Δ*rnd*	RNase D trims tRNA 3′ ends for maturation	SNP	Damaging (PolyPhen-2)
Δ*dld*	Membrane enzyme oxidizing D-lactate to quinones	IN	Premature termination
Δ*acrB*	AcrB is a proton-driven transporter in AcrAB-TolC	SNP	Damaging (PolyPhen-2)
Δ*ybcL*	Periplasmic protein inhibiting neutrophil migration	DEL	Premature termination
Δ*uidB*	Encodes UidB, a transporter for glucuronide import	SV	Premature termination
Δ*dsdC*	A DNA-binding transcriptional regulator	IN	Premature termination
Δ*narJ*	Chaperone for NarG molybdenum cofactor incorporation	IN	Premature termination
Δ*yoeH*	*yoeH* is a pseudogene encoding a nonfunctional transposase	DEL	Disrupts the reading frame—non-functional protein
Δ*recF*	RecF loads RecA, enabling recombination and restart	SV	Premature termination
Δ*rhsC*	A repetitive, hydrophilic protein, involved in cell interactions	SV	Premature termination
Δ*dinB*	*dinB* encodes Pol IV, a polymerase that bypasses DNA lesion	IN	Disrupts the reading frame—non-functional protein^*d*^
Δ*flgN*	Chaperone aids FlgK and FlgL export; prevents aggregation	SV	Premature termination
Δ*hybE*	HybE coordinates hydrogenase 2 assembly, interacting with HybO and HybC	SV	Premature termination
Δ*yjeP*	YjeP (MscM) protects *E. coli* from hypoosmotic shock	IN	Premature termination
Δ*yghD*	Nonfunctional inner membrane protein due to T2SSβ deletions	SV	Premature termination
Δ*yfjR*	YfjR is a transcription factor for phage processes and biofilms	IN	Premature termination
Δ*pgl*	Pgl encodes 6-phosphogluconolactonase, aiding growth and stress responses in *E. coli*	SV	Disrupts the reading frame—non-functional protein
Δ*putA*	PutA is a redox-regulated enzyme that degrades proline and represses the put operon	SV	Disrupts the reading frame—non-functional protein
Δ*amiB*	An amidase that cleaves peptidoglycan, aiding cell division	SNP	Damaging (PolyPhen-2)
Δ*rlmB*	Modifies G2251 of 23S rRNA, protecting it from damage	IN	Premature termination
Δ*yodB*	YodB is a membrane protein with four transmembrane helices	IN	Premature termination
Δ*kdpA*	A subunit of Kdp ATPase complex, facilitating K+ transport	SNP	Premature termination
Δ*appC*	AppC encodes subunit I of cytochrome bd-II, involved in electron transfer	SV	No stop codon—non-functional protein^*e*^
Δ*arpA*	ArpA, linked to neonatal meningitis, contains ankyrin repeats	SNP	Damaging (PolyPhen-2)
Δ*pbpC*	Penicillin-binding protein with minimal impact on peptidoglycan synthesis	SV	Premature termination
Δ*rpmI*	RpmI encodes ribosomal protein L35, regulated by L20	IN	Premature termination
Δ*prpC*	PrpC catalyzes a key step in propionate catabolism, induced by propionate	SV	Disrupts the reading frame—non-functional protein
Δ*yraP*	A lipoprotein aiding cell division and membrane integrity	SV	Premature termination
Δ*qseC*	QseC senses host signals to regulate motility via QseB	DEL	Premature termination
Δ*yhaL*	Uncharacterized protein YhaL	SV	Premature termination
Δ*iaaA*	Isoaspartyl dipeptidase proenzyme	SV	Premature termination
Δ*malS*	MalS is a periplasmic α-amylase that breaks down maltodextrins for nutrient use	SV	No stop codon—non-functional protein
Δ*aroE*	AroE catalyzes shikimate formation in aromatic amino acid biosynthesis	DEL	Premature termination
Δ*ugpQ*	UgpQ hydrolyzes glycerophosphodiesters during phosphate starvation	DEL	Premature termination
Δ*rhsB*	Protein likely involved in competition or stress response	SV	Premature termination
Δ*yidF*	YidF boosts D-serine resistance when overexpressed	SV	Disrupts the reading frame—non-functional protein
Δ*phnG*	PhnG aids phosphonate breakdown in the C–P lyase complex	SV	No stop codon: non-functional protein
Δ*dgcQ*	Makes c-di-GMP, controlling cellulose, motility, and biofilms	IN	Premature termination
Δ*ygfF*	Putative oxidoreductase YgfF	SV	Premature termination
Δ*adk*	It balances AMP/ADP, supporting nucleotide synthesis, growth	SNP	Damaging (PolyPhen-2)
Δ*gpmM*	GpmM is a phosphoglycerate mutase aiding oxidative stress resistance; its loss increases H_2_O_2_ sensitivity	SV	Disrupts the reading frame— non-functional protein
Δ*gltA*	GltA catalyzes the TCA cycle’s first step, converting acetyl-CoA and oxaloacetate to citrate	SV	Disrupts the reading frame—non-functional protein
Δ*fau*	Fau is a 5-CHO-THF cyclo-ligase supporting stress survival and antibiotic tolerance	SV	No stop codon:non-functional protein
Δ*nuoA*	NuoA is a subunit of NADH dehydrogenase I, crucial for electron transport and aerobic growth	SV	Disrupts the reading frame—non-functional protein
Δ*uvrD*	UvrD is a helicase that aids DNA repair, replication, recombination, and persister formation	SV	Disrupts the reading frame—non-functional protein

^*a*^ Gene function information was sourced from EcoCyc (https://ecocyc.org/).

^*b*^ Damaging (PolyPhen-2): the mutation is predicted to be damaging and likely produces a dysfunctional protein by PolyPhen-2.

^*c*^ Premature termination: translation halts early because of stop codons, yielding a usually nonfunctional truncated protein.

^*d*^ Disrupts the reading frame—non-functional protein: deletion or insertion of a specific sequence at the functional site within the coding sequence is likely to disrupt protein activity, resulting in a non-functional protein.

^*e*^ No stop codon—non-functional protein: deletion or insertion within the coding sequence removes the stop codon, producing an abnormally long, likely non-functional protein.

**Fig 5:**
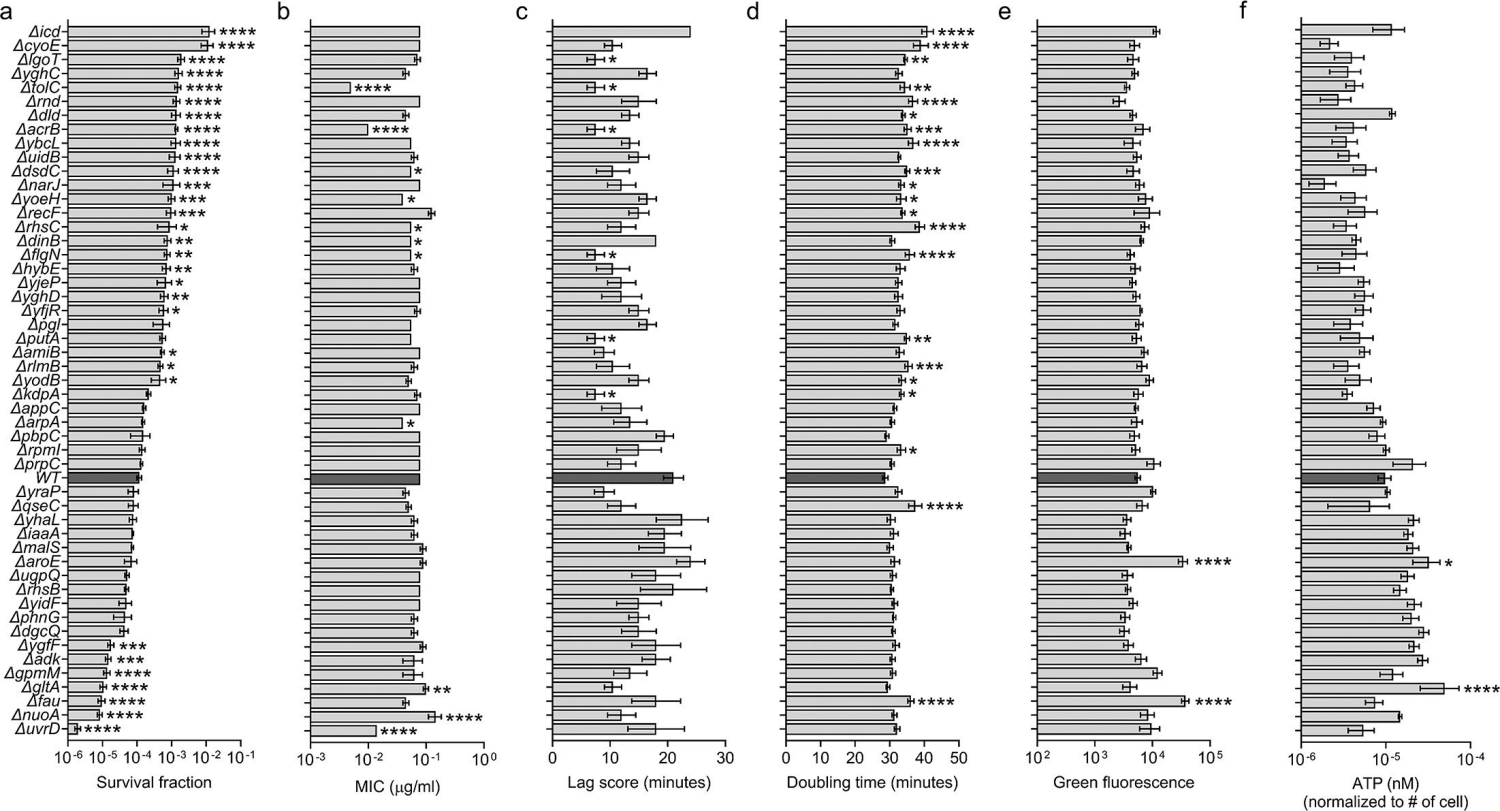
Survival fraction, MIC, fitness factors, and metabolic activities of the single mutants revealed new genetic determinants for tolerance. (**a**) Stationary phase cells of the designated individual single mutants were exposed to ofloxacin (5 µg/mL) after diluting 100-fold in LB media for 7 h. Treated cells were collected, washed, and plated on LB agar plates to enumerate the CFU levels. *n* = 4. (**b**) Indicated single mutants were diluted to have ~10^8^–10^9^ cells/mL and spread on a circular LB agar plate. Agar plates were dried for 20 min next to the flame. Ofloxacin ETEST strips were placed on the dried agar plates and incubated for 16 h to determine the minimum inhibitory concentrations. *n* = 3. (**c**) Lag scores of the indicated individual mutant strains were calculated using the growth curves (Fig. S5). *n* = 4. (**d**) Doubling times of the indicated individual strains were calculated using the exponential phase of the growth curves (Fig. S5). *n* = 4. (**e**) Stationary phase cells of individual single mutants were stained with RSG dye and analyzed with a flow cytometer to determine the metabolic state of the cells (Fig. S6). *n* = 4. (**f**) Stationary phase cells of the indicated strains were collected to measure the intracellular ATP concentrations (see Materials and Methods). A flow cytometer was used to count the cell number for normalization purposes. *n* = 4. Statistical analysis was performed between the WT and single mutants using one-way ANOVA with Dunnett’s post-test. *P < 0.05, **P  <  0.01, ***P  <  0.001, and ****P  <  0.0001. Data corresponding to each time point represent the mean value ±  standard error.

**Fig 6:**
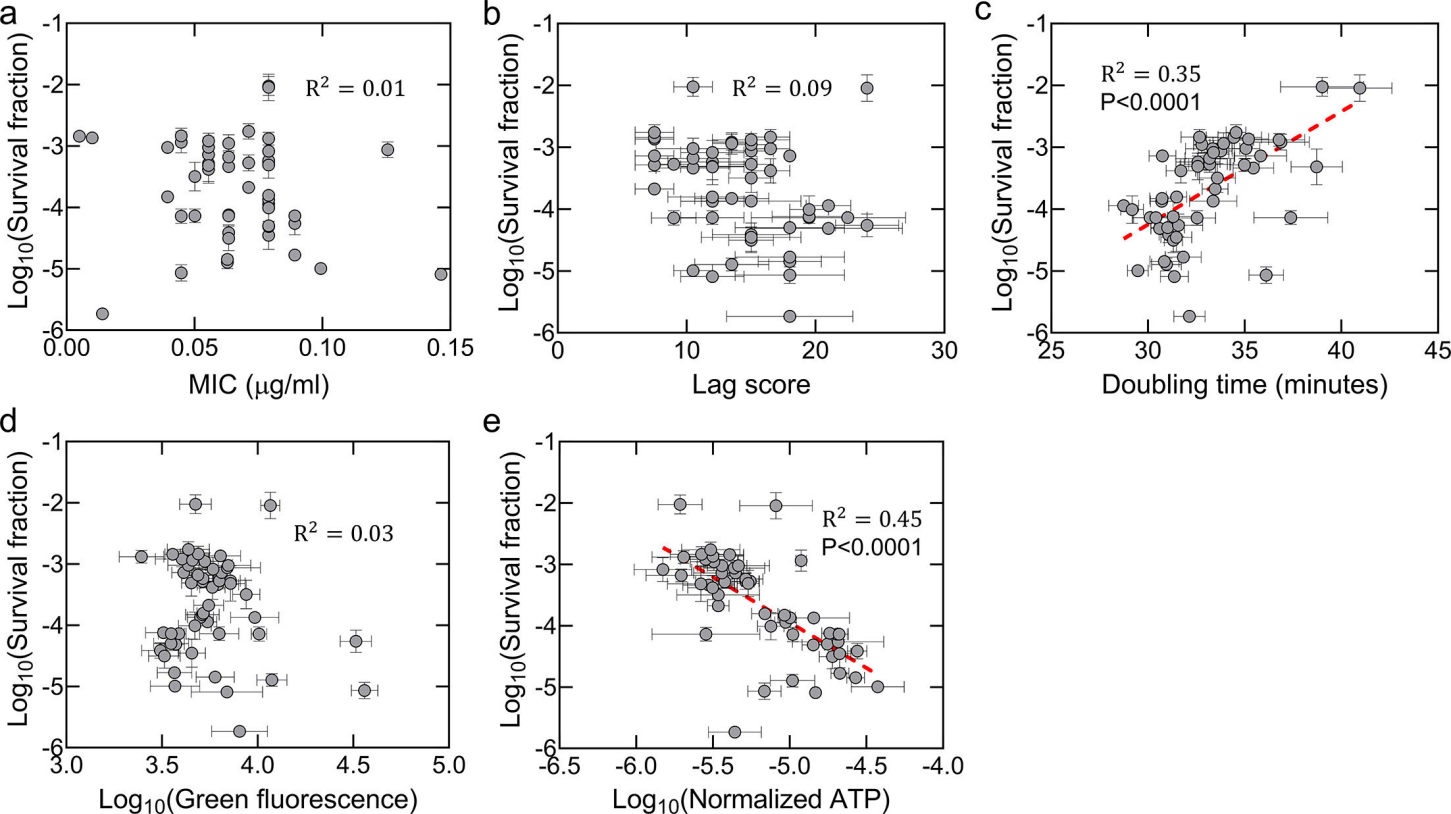
Correlation between survival fractions, fitness factors, and metabolic parameters of knockout strains. All data are from Fig. 5B through f. Pearson correlation analysis was performed between survival fractions and (**a**) MIC, (**b**) lag scores, (**c**) doubling times, (**d**) cellular redox states, and (**e**) ATP levels of single knockout strains. *P* values (for statistically significant correlations) and *R*^2^ values (Pearson correlation) are shown on the graphs. Data are presented as mean ± standard error.

Based on these predictions, which suggest that many of the mutations lead to dysfunctional proteins, we opted to delete each of the 50 candidate genes individually in the parental *E. coli* MG1655 background. We then evaluated their survival fractions, MIC levels, and fitness parameters to gain insights into the roles of these genes in shaping the phenotypic variation observed in the evolved populations. Since our background *E. coli* MG1655 strain, used for gene deletions, did not have the mCherry expression cassette, we could not determine competition or non-growing cell scores. However, we were still able to determine the critical fitness parameters, such as doubling times, lag scores, and other metabolic properties (Fig. S6 and S7). In the case of survival fraction, 24 single knockout strains (some of them are Δ*icd*, Δ*cyoE*, Δ*lgoT*, Δ*yghC*, Δ*tolC*, Δ*rnd*, Δ*dld*, Δ*acrB*, Δ*ybcL*, Δ*uidB*, Δ*dsdC*, Δ*narJ*, Δ*yoeH*, Δ*recF*, Δ*rhsC*, Δ*dinB*, Δ*flgN*, Δ*hybE*, Δ*yjeP*, and Δ*yghD*) displayed enhanced ofloxacin tolerance compared to the wild-type control strain (Fig. 5a). A smaller set of strains (Δ*ygfF*, Δ*adk*, Δ*gpmM*, Δ*gltA*, Δ*fau*, Δ*nuoA*, and Δ*uvrD*) exhibited notably reduced ofloxacin tolerance in contrast to the wild-type control (Fig. 5a). Several mutants, including Δ*tolC*, Δ*acrB*, and Δ*uvrD*, exhibited significantly lower MIC levels, whereas Δ*nuoA* exhibited slightly higher MIC levels compared to the wild type (Fig. 5b). However, no correlation was observed between the MIC levels and the survival fractions of these mutant strains (Fig. 6a). Simultaneous mutations that may increase or decrease tolerance or resistance could explain the observed fluctuations in survival rates and MICs of the mutant strains.

When we analyzed the fitness factors of the generated knockout strains, we found that strains with higher survival fractions tended to have slightly higher doubling times and lower ATP values (Fig. 5d and f) and observed a moderate correlation between survival fractions and these two parameters (Fig. 6c and e). However, we did not detect any clear trend or correlation between survival fractions and the redox levels of the strains (Fig. 5e and 6d) or lag scores (Fig. 5c and 6b). Nevertheless, our data clearly show that knockout strains exhibit diverse fitness characteristics irrespective of their tolerance or resistance levels (Fig. 5a through f), and correlations between fitness and survival are not always strong or consistent (Fig. 3 and 6).

## DISCUSSION

In this study, we employed ALE experiments, phenotypic profiling, whole-genome sequencing, and gene knockout experiments to examine how *E. coli* adapts to cyclic ofloxacin exposure. Our findings provide several key insights into antibiotic adaptation: (i) we report the emergence of mutants with high ofloxacin tolerance but reduced MIC values, a phenotype rarely studied in the literature; (ii) we identify novel genetic contributors to adaptation, including *cyoE, lgoT, yghC, rnd, uidB, dsdC, narJ, yoeH, flgN, hybE, yjeP, and yghD*, many of which are involved in respiration, metabolism, membrane transport, or stress response; (iii) we show that key fitness metrics, such as lag phase, redox activity, and ATP levels, do not consistently correlate with tolerance or resistance, challenging classical models of fitness trade-offs; and (iv) our data suggest that bacterial adaptation under intermittent antibiotic stress follows diverse, non-convergent evolutionary paths, highlighting the complexity of resistance development in fluctuating environments.

ALE experiments provide us with valuable insights into how bacteria develop resistance, especially under the rapid evolutionary pressures of antibiotic treatment (16, 17, 19, 21, 45). In our study, seven out of eight *E. coli* populations (S1 and S3–S8) evolved increased survival during ofloxacin exposure, while one population, S2, did not show enhanced tolerance after 22 days and retained an MIC similar to the parental wild-type strain. Interestingly, whole-genome sequencing revealed multiple mutations in S2, yet its phenotype remained largely unchanged. This may indicate that the mutations it acquired did not affect key pathways involved in fluoroquinolone tolerance or resistance. Also, S2 may have followed a slower evolutionary trajectory, requiring additional time to accumulate mutations with functional significance. Supporting this, in our separate ALE experiments conducted without kanamycin (Fig. S3a), we observed a comparable case in which a strain maintained low survival levels until day 18, followed by an increase. Such outcomes are not unexpected, as evolution under antibiotic stress is inherently stochastic and can yield diverse adaptive results even under identical conditions. These differences may stem from factors such as mutation timing, the specific genes affected, and subtle environmental variations at the microscale (72, 73). Notably, we also found no correlation between survival fractions and MIC levels, suggesting that tolerance and resistance may evolve independently in cyclic ALE experiments. Under selective pressure, bacteria appear capable of adopting multiple survival strategies, some allowing persistence at high antibiotic concentrations without an increase in MIC.

We demonstrated that the 10 randomly chosen colonies from each sample consistently had similar survival rates and MIC levels. This suggests that exposing the cells to antibiotics repeatedly enriched mutant strains with consistent traits within each sample. This uniformity might come from a few mutant lineages exhibiting greater fitness and survival characteristics; the cyclic pressure from ofloxacin during the ALE process may favor these lineages, eventually leading to the dominance of these specific traits within each sample. However, despite this consistency within individual samples, there was significant diversity across the eight samples. It is obvious that different mutant lineages within each sample follow unique evolutionary paths. Even though all samples started from the same strain, random mutations during the ALE process could have created genetic diversity among the samples. These genetic differences should explain the varying responses to ofloxacin, resulting in different survival rates and MIC levels across samples. Although our study followed an ALE framework, we acknowledge that using the DNA-damaging antibiotic ofloxacin likely increased mutation rates, introducing elements of forward genetic screening. This may explain the broad genetic diversity observed among evolved populations. Nonetheless, this approach reflects real-world conditions where repeated antibiotic exposure can drive both selection and mutagenesis, offering valuable insight into the evolution of tolerance and resistance, given the fact that quinolone antibiotics are among the commonly prescribed antibiotic classes worldwide (74, 75).

We explored fitness traits of the samples (S1–S8) to establish if there is a trade-off between antibiotic tolerance/resistance and these traits. Except for S2, all samples had slightly longer doubling times than the control. In co-culture environments, samples S4 and S8 showed notable growth suppression, indicating a decreased capacity to compete with parental cells. There seems to be a connection between survival and competitive advantage, as evidenced by the moderate correlation found between survival fractions and doubling times or competition scores of the samples. However, we found no evidence of a correlation between bacterial survival and other fitness parameters linked to non-growing cell counts, lag scores, and metabolic processes. Previous studies have identified the lag phase as a critical factor in the evolution of antibiotic-resistant strains (16, 50), and traditional models suggest that resistance mutations often impose fitness costs (51, 52, 76). While we fully agree with these perspectives, it is important to emphasize that these traits are contingent on the environmental conditions that organisms encounter. For instance, cyclic ALE experiments might select for mutations that confer resistance with minimal fitness trade-offs, as the recovery phase allows resistant mutants to compete and proliferate. This process can lead to the selection of optimized resistance mechanisms, favoring mutations that do not drastically impact metabolic efficiency or growth capabilities. The alternating selective pressures of cyclic ALE mimic real-life fluctuating environments, and bacteria evolving under these conditions may develop more robust and adaptable resistance mechanisms that perform well under both stress and non-stress conditions.

We carried out whole-genome sequencing to get further insight into genetic backgrounds of the samples that have diverse fitness traits and antibiotic resistance and tolerance properties. We do not think that the evolution of resistance appears to follow a consistent pathway or mechanism, even though a few mutations in the same genes occur in different samples. Common mutations found in samples for particular genes, such as *icd, cyoE*, *rhsC*, and *adk*, represent potential targets for further investigation. We are aware that each sample has diverse mutations in numerous genes, and these mutations may eliminate, enhance, or completely change the functions of these genes. Given the combinatorial impact of all these mutations on antibiotic tolerance and resistance, it is very challenging to gain a mechanistic understanding from these diverse mutational landscapes. Nevertheless, we deleted individual genes, including those exhibiting the highest frequency of mutations, to assess their effects on cellular antibiotic resistance, tolerance, and fitness characteristics. When compared to wild-type strains, a significant number of knockout strains exhibit increased ofloxacin tolerance, as expected. While our research supports the prior findings that several of these knockout strains, including metabolic mutations, enhance bacterial survival (21), we also found new genes potentially associated with antibiotic tolerance and/or resistance, including *cyoE, lgoT, yghC, rnd, udiB, dsdC, narJ, yoeH, flgN, hybE, yjeP, yghD, yfjR, amiB, adk,* and *ygfF*. Many of the identified genes are associated with critical cellular functions, including respiration and energy metabolism (*cyoE*, *narJ*, and *hybE*) (77–79), motility and cell envelope remodeling (*flgN* and *amiB*) (80, 81), metabolism and nucleotide balance (*adk* and *uidB*) (82, 83), transcriptional regulation and stress response (*dsdC*, *yoeH*, *yfjR*, and *yjeP*) (84–87), and membrane transport or efflux pumps (*lgoT*, *yghC*, *rnd*, and *ygfF*) (88–91) (Table 1). Some genes remain uncharacterized (*yghD*) (92). While the functions of many of these genes are known (Table 1), their specific roles in antibiotic resistance and tolerance remain unclear, presenting promising avenues for future research. Additionally, we observed a few knockout strains Δ*uvrD,* Δ*nuoA,* and Δ*gltA,* with decreased tolerance, which is consistent with previous studies (93, 94). The concurrent emergence of mutations that may either enhance or reduce tolerance could account for the observed fluctuations in the survival fractions of the mutant strains.

Many fitness parameters of the knockout strains do not show a clear trend or correlation with survival fractions, although some knockout strains with greater survival fractions tend to have slightly higher doubling scores and lower ATP values. The observed variations in metabolic activities among the knockout strains may indicate that bacterial cells may use diverse metabolic strategies to deal with antibiotic-induced stress. In fact, our whole-genome sequencing revealed the enrichment of mutations in several metabolic genes, such as *icd*, *nuo,* and *gltA*. This is in line with recent research that highlights the possible contribution of metabolic adaptations to antibiotic tolerance. Van den Bergh’s group (95) investigated genomic alterations in *E. coli* strains— including uropathogenic UTI89—that were exposed to antibiotics periodically and observed mutations in the genes of the *nuo* operon, a critical element of *E. coli* energy metabolism. Additionally, the work conducted by Lopatkin’s group (21) identified hitherto unidentified metabolic genes (*icd, gltD,* and *sucA*) that are implicated in the development of antibiotic resistance in *E. coli* cells exposed to a variety of antibiotics. They further supported these results by analyzing a huge library of 7,243 *E. coli* genomes from NCBI Pathogen Detection, showing that comparable mutations are present in clinical *E. coli* infections (21).

The deletion of the *icd* gene has been repeatedly shown to increase the antibiotic tolerance of bacterial cells by various studies (21, 96, 97). Although the identification of mutations in the *icd* gene in most samples, including S2, in our study might imply a conserved evolutionary mechanism, the mutations in this gene alone do not fully explain the observed tolerance, as S2 does not exhibit increased tolerance or resistance. This may imply the collective effects of the multiple mutations in shaping the evolutionary pathways of resistant or tolerant strains. Additionally, our data show that not all metabolic gene knockout strains carry significant fitness costs, while the *icd* knockout exhibits such costs. This is also supported by several recent studies (96, 97). This phenomenon could give a survival advantage to bacterial cells, as they may acquire metabolic mutations to increase their tolerance without experiencing significant fitness costs.

In this study, we used ofloxacin as the antibiotic in our ALE experiments, which is known to commonly induce mutations in genes such as *gyrA*, *gyrB*, *parC*, and *parE* (98–100). However, these mutations were largely absent in our evolved strains, with the exception of *gyrB* mutations observed in S4 and S8. Antibiotic type, cell growth conditions, and the design of ALE experiments are critical factors that influence the trajectory of resistance evolution. In most published studies, antibiotic concentrations are gradually increased and maintained throughout the experiment to impose sustained selective pressure (19, 32, 33, 35). This continuous exposure typically selects for mutant strains with elevated MICs, often through mutations in drug target genes, such as *gyrA*, *gyrB*, *parC*, and *parE,* in the case of quinolone resistance (101–103). In contrast, our experimental design employed intermittent high-dose ofloxacin exposure followed by recovery periods in antibiotic-free media. This cyclic regimen likely altered the nature of selection, favoring metabolic adaptations rather than mutations in target-site genes. During antibiotic-free recovery, strains must compete under normal growth conditions, where target-site mutations (particularly in essential genes like *gyrA*) could reduce cellular fitness and growth, making such mutants less competitive. This interpretation aligns with previous reports showing that mutations in essential genes such as *gyrA* can impair growth (104, 105).

In conclusion, in this study, we demonstrated how bacterial cells acquire complex resistance or tolerance mechanisms to survive unfavorable conditions. Although we observed a few common mutations across different evolved samples, we did not find a clear pattern of evolution leading to a conserved pathway or mechanism. Our study, which uncovers the complex relationship between antibiotic tolerance, genetic mutations, and bacterial fitness, adds to our understanding of bacterial adaptation mechanisms, which may help guide the development of therapeutic interventions to address the heterogeneous nature of antibiotic resistance.

## MATERIALS AND METHODS

### Bacterial strains and plasmids

In this study, we used *Escherichia coli* K-12 MG1655 and the MO strain. Unless noted otherwise, all the knockout strains in *E. coli* MG1655 were generated during the study. *E. coli* MO strain harbors red fluorescence (*mCherry* gene) expression system in the genome (57, 65). pUA66-*gfp* expression system was used with the kanamycin-resistant gene as a selection marker. Both *mCherry* and *gfp* are isopropyl β-D-1-thiogalactopyranoside inducible and tightly regulated by *T5* and *LacI^q^* as promoter and repressor, respectively. A complete description of bacterial strains and plasmids used in this study is listed in Table S1. The primers used to generate knockout strains are tabulated in Tables S3 and S4. *mCherry* and *gfp* expressions were used to monitor the cell proliferation at the single-cell level with flow cytometry. Fluorescent protein overexpression through chromosomally integrated *mCherry* and plasmid pUA66-*gfp* did not alter the antibiotic sensitivity of *E. coli* cells. An empty vector of pUA66 plasmid (without *gfp* gene) was used as a control throughout the study.

### Media, chemicals, and culture conditions

Unless otherwise noted, chemicals utilized in this study were purchased from Fisher Scientific (Atlanta, GA, USA), VWR International (Radnor, PA, USA), or Sigma-Aldrich (St. Louis, MO, USA). To grow the bacteria in liquid media, Lysogeny Broth was prepared from its components, which include yeast extract (5 g per 1 L DI water), tryptone (10 g per 1 L DI water), and sodium chloride (10 g per 1 L DI water). LB agar (40 g premixed per 1 L DI water) was used as solid media to grow the bacteria whenever required. IPTG (1 mM as final concentration) was used to overexpress fluorescent proteins (mCherry or GFP) in *E. coli* (57, 67). For plasmid retention and antibiotic selection marker, 50 µg/mL kanamycin was used. To perform an *in vitro* clonogenic survival assay on *E. coli* cells, 5 µg/mL ofloxacin was used. Phosphate-buffered saline solution was used to remove chemicals and antibiotics from the liquid cell cultures before spotting them on the agar plate to enumerate the surviving cells. Ultra-pure distilled water was used to prepare chemical and antibiotic solutions as well as growth media. The NaOH solution was used to dissolve ofloxacin in water, where the final concentration of NaOH became 0.06 mM. Bacterial growth media were sterilized by autoclaving at high temperature (121°C) and pressure (15 psi). Chemical and antibiotic solutions dissolved in water were sterilized using a 0.2 µm polyether sulfone membrane filter. The chemicals used in this study are listed in Table S5. FlowJo Software (Tree Star Inc., Ashland, OR, USA) was used to analyze all the flow cytometry samples in this study. Overnight pre-cultures were prepared by inoculating 2 mL of LB media in 14 mL Falcon tubes with cells from a frozen (−80°C) cell stock and cultured at 37°C with shaking (250 rpm) for 16 h.

### Adaptive laboratory evolution technique

To enrich the *E. coli* cultures with tolerant or resistant cells, the adaptive laboratory evolution technique was adopted. Overnight pre-cultures of *E. coli* MO strain harboring pUA66-EV were diluted 100-fold (~3–5 × 10^7^ cells/mL) in 2 mL LB media in a 14 mL Falcon tube, followed by treatment with 5 µg/mL of ofloxacin and incubated at 37°C with shaking (250 rpm). For plasmid maintenance, kanamycin (50 µg/mL) was added to the LB media (67). At the end of the 7-h treatment, 1 mL of treated cells was washed three times with PBS (1×) by centrifugation (13,300 rpm or 17,000 × *g*) to remove antibiotics. After the final washing step, pelleted cells were inoculated in 2 mL LB media and grown in a shaker as an overnight pre-culture for the subsequent day. To determine the number of surviving cells after antibiotic treatment, the remaining 1 mL of treated cells was washed as described above. At the final washing step, 900 µL supernatant was removed, and pelleted cells were resuspended in the remaining 100 µL PBS. Resuspended cells were serially diluted in 90 µL PBS. Ten microliters of cell suspension was spotted on the agar plate to enumerate the surviving cells. Before ofloxacin treatment, 10 µL cell suspensions were serially diluted in 90 µL PBS and spotted on the agar plate to count the initial number of cells. Agar plates with cells were incubated at 37°C for 16 h to allow *E. coli* colony formation. This cyclic intermittent antibiotic treatment was carried out for 22 days without interruption. After 22 days, 25% glycerol cell stocks were prepared for the evolved cell samples and stored at −80°C for future assays.

To confirm the homogeneity of ofloxacin resistance/tolerance among the individual evolved cell samples, frozen cell samples were streaked on LB agar plates containing 50 µg/mL kanamycin (to avoid potential contamination). Agar plates were incubated at 37°C for 16 h. Ten random colonies from each evolved cell sample were picked (total 88 colonies: 10 from each of the eight samples and 8 from the parental strain) and cultured in LB media in a 96-well plate format at 37°C with shaking. Breath-easy Membrane (Catalog# NC3356703, Fisher Scientific, USA) was used to cover the 96-well plate. Once the cultures reached the stationary phase (*t* = 16 h), 25% glycerol cell stock of each culture was prepared and stored at −80°C for subsequent assays.

### Cell growth assay and calculations of lag and doubling time scores

Compared to the parental strain, cell growth was measured in optical density to assess the growth trend of the eight evolved samples. The growing cultures’ optical density (OD_600_) was monitored at 600 nm wavelength using a plate reader (Varioskan LUX Multimode Microplate Reader, Thermo Fisher, Waltham, MA, USA) for 24 h. Cell growth assay cultures were prepared by diluting overnight pre-cultures 100-fold (~3–5 × 10^7^ cells/mL) in 2 mL LB media in 14 mL Falcon tubes and grown at 37°C with shaking (250 rpm). At designated time points, 300 µL of cell suspension was placed in the 96-well flat-bottom plates, and the OD_600_ was measured with the plate reader. The lag score, representing the time bacteria take to initiate growth in a specific medium, was calculated using time-dependent growth data and determined based on the methodology outlined in previous studies (63, 64). Doubling time is the duration it takes for bacteria to double their population size through binary fission. We calculated the doubling time using data from the exponential growth phase with the following formula:


$$N=N_{0}2^{\left(t-t_{0}\right)/t_{d}},$$

where *N*_0_ is the initial number of cells when the exponential phase starts at *t*_0_, *N* is the number of cells at time *t*, and *t*_*d*_ is the doubling time.

### Antibiotic tolerance assay

Overnight pre-cultures were diluted 100-fold (~3–5 × 10^7^ cells/mL) in 2 mL LB media in a 14 mL Falcon tube and immediately treated with antibiotics (ampicillin, gentamycin, and ofloxacin) and cultured at 37°C with shaking (250 rpm). Kanamycin (50 µg/mL) was added to the culture to retain the plasmid. To determine the initial number of cells before antibiotic treatment, 10 µL of cell suspension was serially diluted in 90 µL of PBS and spotted on the agar plate. At the designated time point after treatment (i.e*.,* 7-h treatment), 1 mL of treated cells was washed twice with PBS (1×) to reduce the antibiotic concentration at the sub-MIC level by centrifugation (13,300 rpm or 17,000 × *g*). After the final centrifugation, 900 µL supernatant was removed, and pelleted cells were resuspended in the remaining 100 µL PBS. Resuspended cells were serially diluted in 90 µL of PBS to facilitate colony counting after plating on agar. Then, serially diluted cell suspensions were spotted on the agar plates to enumerate the CFU, as described above. To grow the *E. coli* colonies, agar plates were incubated at 37°C for 16 h.

### Non-growing cell detection assay

To determine the level of non-growing cells in the evolved samples and the parental strain (*E. coli* MG1655 MO strain harboring pUA66-empty vector), the IPTG-inducible red fluorescent protein (mCherry) expression system was used. To obtain the red fluorescence protein (mCherry) positive cells, 1 mM IPTG was added to the overnight pre-cultures. mCherry-positive cells (1 mL) from the overnight pre-cultures were washed with PBS three times to remove IPTG and resuspended in 1 mL sterile LB media. Washed cells were diluted 100-fold in 2 mL LB broth without the inducer (IPTG) in 14 mL Falcon tubes and cultured at 37°C with shaking (250 rpm). At designated time points (*t* = 0, 1, 2, and 3 h), cells were diluted in PBS, and their red fluorescent protein (mCherry) levels were measured through a flow cytometer (NovoCyte Flow Cytometer, NovoCyte 3000RYB, ACEA Biosciences Inc., San Diego, CA, USA). We note that cells were always diluted in PBS to achieve a cell density of ~10^6^–10^7^ cells/mL for flow analysis. At the beginning of cell culturing without inducer (*t* = 0), cells expressed high levels of red fluorescence. Later, once cells started to proliferate, due to protein dilution, cells gradually lost their red fluorescence protein levels. Finally, at *t* = 3 h, a small subpopulation of the culture, consisting of non-growing cells, still exhibited high levels of red fluorescent protein due to a lack of cell division. The fractions of these non-growing cells were determined by flow cytometry. The excitation wavelength for red fluorescence was 561 nm, and a 615/20 bandpass filter received the fluorescence signals.

### Competition assay

For the competition assay, equal numbers of cells from both the parental strain (*E. coli* MG1655 MO strain harboring pUA66-*gfp*) and an evolved strain (S1, S2, S3, S4, S5, S6, S7, and S8) were diluted in 2 mL of LB media from overnight pre-cultures and grown at 37°C with shaking (250 rpm) for 24 h. To induce red (mCherry) and green (GFP) fluorescent proteins, 1 mM IPTG was added to the pre-cultures and competition cultures. We note that parental strain harboring plasmid expressing GFP was used to monitor their growth through a flow cytometer. During the co-culturing, at designated time points (*t* = 0, 4, 8, and 24 h), cells were collected and diluted in PBS to be analyzed through a flow cytometer. Cells were diluted in PBS to reach a cell density of around 10^6^–10^7^ cells/mL for the flow cytometry analysis. The excitation wavelength for green fluorescence was 488 nm, and a 530/30 bandpass filter collected signals. The excitation wavelength for red fluorescence was 561 nm, and a 615/20 bandpass filter received the fluorescence signals. To maintain the plasmid, kanamycin (50 µg/mL) was added to the LB medium.

### Minimum inhibitory concentration determination

Overnight pre-cultures of parental strain and evolved strains were prepared as described above. When necessary, kanamycin (50 µg/mL) was added to the culture to retain the plasmid. Agar plates were prepared using circular Petri dishes (100 × 15 mm, Fisher Scientific, catalog# FB0875712) into which approximately 15–20 mL of melted LB agar was poured. Overnight pre-cultures of each sample were diluted (~5 × 10^8^ cells/mL) in 1 mL PBS and spread with an L-shape sterile spreader on the agar plate. Inoculated plates were dried at room temperature (~25°C) next to a flame for 15 min. Ofloxacin (OFX) MIC Test Strip (Fisher Scientific, catalog# 22-777-876) was placed in the dried agar plate and incubated at 37°C for 20 h. We note that the MIC Test Strip contained known concentration gradients of ofloxacin ranging from 0.002 to 32 µg/mL.

To determine the MIC levels of 10 randomly selected colonies from each evolved sample (total 88 colonies), we performed a two-fold microdilution in a 96-well format (106). The MIC for each colony was calculated as the average of the lowest antibiotic concentration with no visible bacterial growth and the highest concentration with bacterial growth. Since MIC is a transitional point between growth and no growth, the exact concentration at which inhibition truly occurs likely falls between these two values. Averaging these provides a reasonable approximation that avoids underestimating or overestimating the MIC.

### ATP measurement

Intracellular ATP levels in the mutant strains were quantified using the BacTiter-Glo Microbial Cell Viability Assay Kit (Catalog# G8230, Promega Corporation, Madison, WI, USA), following the manufacturer’s instructions. Fresh LB medium was used to determine background luminescence. Standard curves were generated by measuring luminescence from known concentrations of ATP, prepared through serial dilutions of rATP (Promega Catalog# P1132) in fresh LB medium.

### Redox Sensor Green dye staining

Bacterial reductase activity was assessed using RSG dye (Catalog# B34954, Thermo Fisher). Overnight cultures were diluted in 0.85% NaCl to a final density of ~ 10⁶–10⁷ cells/mL and stained with 1.0 µM RSG in 5 mL round-bottom flow cytometry tubes (12 × 75  mm). After brief vortexing (~10 s), samples were incubated at 37°C in the dark for 10 min. Fluorescence was measured using a NovoCyte 3000RYB flow cytometer (ACEA Biosciences, San Diego, CA, USA) at a slow sample flow rate (14 µL/min; core diameter 7.7 µm), with a constant sheath flow of 6.5 mL/min. Cells were excited at 488 nm, and green fluorescence was detected using a 530/30 bandpass filter.

### Next-generation sequencing

To identify the genetic perturbations on the genome of the evolved cells, next-generation sequencing was performed in GeneWiz (South Plainfield, NJ, USA). Overnight pre-cultures of the parental and evolved strains were diluted 100-fold (~3–5 × 10^7^ cells/mL) in 2 mL LB media with 50 µg/mL kanamycin in 14 mL Falcon tubes and grown at 37°C with shaking (250 rpm). Late-exponential phase (OD_600_ ~ 1.0) cells were collected and washed twice with PBS (1×) by centrifugation (13,300 rpm or 17,000 × *g*). At least 100 µL of cell pellet was collected in Eppendorf tubes after the final washing step. Eppendorf tubes containing pelleted cells of the individual sample were immediately immersed in a dry ice-ethanol bath (~−80°C) for 10 min. Frozen cell pellets were stored at −80°C before being shipped to GeneWiz. Notably, the wild-type parental strain was used as the background genome sequence control. Detailed sequencing methods obtained from GeneWiz are outlined below:

#### Extraction

DNA extractions were performed on cell pellets using the Qiagen QIAmp DNA Kit as per the manufacturer’s recommendation.

#### Library preparation

Genomic DNA was quantified using the Qubit 2.0 Fluorometer (Life Technologies, Carlsbad, CA, USA). The DNA integrity was checked with ~1% agarose gel with 50–100 ng sample loaded in each well. Samples were then chosen for library preparation based on the QC results. The NEBNext Ultra II DNA Library Prep Kit for Illumina, including clustering and sequencing reagents, was used throughout the process following the manufacturer’s recommendations. Briefly, the genomic DNA was fragmented by acoustic shearing with a Covaris S220 instrument. Fragmented DNA was cleaned up and end repaired. Adapters were ligated after adenylation of the 3′ ends, followed by enrichment by limited-cycle PCR. DNA libraries were validated using a DNA 1000 Chip on the Agilent 2100 Bioanalyzer (Agilent Technologies, Palo Alto, CA, USA) and were quantified using the Qubit 2.0 Fluorometer.

#### Sequencing

The DNA libraries were also quantified by real-time PCR (Applied Biosystems, Carlsbad, CA, USA) and clustered onto a flow cell on the Illumina MiSeq instrument according to the manufacturer’s instructions. The sample was sequenced using a 2 × 150 paired-end configuration. Image analysis and base calling were conducted by the MiSeq Control Software on the MiSeq instrument. Raw sequencing data (.bcl files) generated from Illumina MiSeq were converted into fastq files and de-multiplexed using Illumina’s bcl2fastq software. One mismatch was allowed for index sequence identification.

#### Data analysis

Sequence data were trimmed to remove possible adapter sequences at the 3′ end and nucleotides with poor quality before analysis using CLC Genomics Server. Trimmed sequence reads were aligned to the reference genomes for *Escherichia coli*. (i) Mapping consensus sequence was generated for each sample, and a FASTA file was created for the mapping consensus sequence. A BAM file and its index file were generated for each sample. (ii) SNP/INDEL detection was performed using the probabilistic model with the CLC Genomics Server program. (iii) A mapping and data coverage summary report was generated for each sample.

### *In silico* methods for predicting protein functionality

To evaluate the potential functional consequences of mutations identified in strains evolved through ALE, we applied computational tools to analyze three major mutation types observed in our whole-genome sequencing data: INDELs, SVs, and SNPs.

For INDELs and SVs, we first introduced the observed mutations into the corresponding DNA sequences at the appropriate genomic positions. The mutated sequences were then translated into protein sequences using the online tool Translate (ExPASy). In many cases, these mutations introduced premature “stop” codons, resulting in truncated protein products likely to be non-functional. In some instances, the native stop codon was eliminated, leading to abnormally long protein sequences lacking a termination signal, also indicative of potential loss of function.

For SNPs, we employed Polymorphism Phenotyping v2 (PolyPhen-2), an online tool that predicts the potential impact of amino acid substitutions on protein structure and function (71). PolyPhen-2 utilizes a combination of sequence conservation, structural features, and comparative evolutionary analyses to assess the likelihood that a given missense mutation is damaging. It generates a probability score and classifies mutations into categories such as benign, possibly damaging, or probably damaging based on a naive Bayes classifier trained on curated data sets. The prediction accounts for multiple factors, including the location of the mutation in conserved domains, changes in amino acid properties, and the potential effect on protein stability and interactions.

### Statistical analysis

To generate a biphasic kill curve, we applied a nonlinear model (19, 68).


$${log}_{10}n_{i}={log}_{10}\left\{\left(n_{0}-p_{0}\right)e^{-k_{n}t}+p_{0}e^{-k_{p}t}\right\},$$

where *n*_0_ is the initial cell count (CFU/mL), *p*_0_ is the initial persister count (CFU/mL), *k*_*n*_ and *k*_*p*_ are the killing rates of normal and persister cells, respectively, *t* is time (h), and *n*_*i*_ is the number of surviving cells (CFU/mL) at time *t*. At least four biological replicates were performed for each condition except the generation of evolved cells. Correlation analysis was performed using Pearson correlation methods, depending on the context. To assess the statistical significance between control and treatment groups, a one-way ANOVA test with Dunnett’s post-test was performed, where the threshold value of *P* was chosen as *P < 0.05, **P < 0.01, ***P < 0.001 and ****P < 0.0001. Average ± standard error was used to present all the data points in the linear graphs. In the case of flow diagrams, a representative figure from among all the replicates is presented in this article, while other replicates showed a similar trend.

## Data Availability

All relevant data are provided in the main figures or supplemental files. The raw data have been deposited in FigShare, https://doi.org/10.6084/m9.figshare.27935040.v1, and the genomic sequencing data have been deposited in ArrayExpress, https://www.ebi.ac.uk/biostudies/arrayexpress/studies/E-MTAB-14682.

## SUPPLEMENTAL MATERIAL

Supplemental Material msystems.00713-25-s0001.pdf Figures S1 to S7; Tables S1 to S5.

ASM does not own the copyrights to Supplemental Material that may be linked to, or accessed through, an article. The authors have granted ASM a non-exclusive, world-wide license to publish the Supplemental Material files. Please contact the corresponding author directly for reuse.
