# Supplementary material for: The diverse phenotypic and mutational landscape induced by fluoroquinolone treatment
Source: mSystems. 2025 Jul 31;10(8):e00713-25. doi: 10.1128/msystems.00713-25 (PMC12363166; doi:10.1128/msystems.00713-25)
Supplement: Supplemental Material — Figures S1 to S7; Tables S1 to S5. [file msystems.00713-25-s0001.pdf]

Supplementary Information

The Diverse Phenotypic and Mutational Landscape Induced by Fluoroquinolone Treatment

Sayed Golam Mohiuddin, Pouria Kavousi, Diego Figueroa, Sreyashi Ghosh, and Mehmet A. Orman*

William A. Brookshire Department of Chemical and Biomolecular Engineering,

University of Houston, Houston, TX 77204-4004

*Corresponding author: morman@central.uh.edu

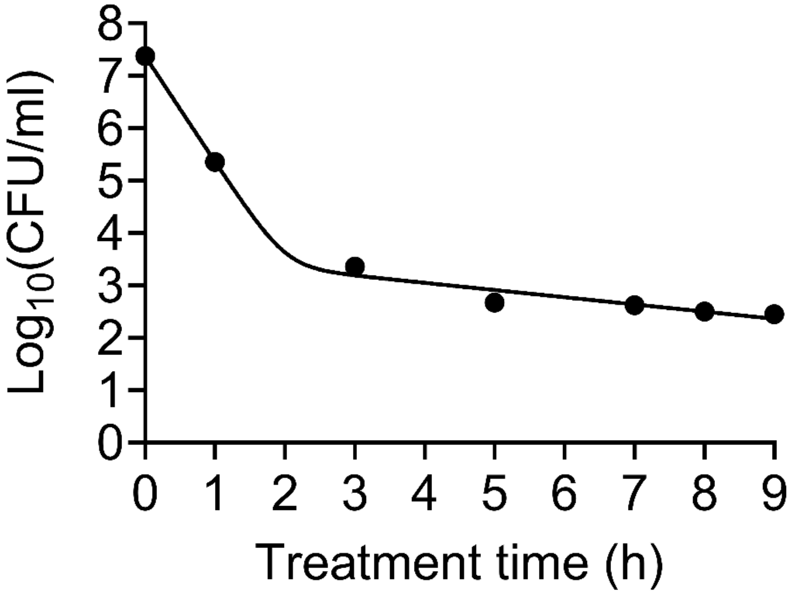

Fig. S1. Biphasic kill curve. Stationary-phase *E. coli* MG1655 MO cells (16 h culture) harboring the pUA66-empty vector were diluted 1:100 in LB and exposed to ofloxacin (5 µg/ml) for 9 h. At specified time points, cells were harvested, washed with PBS, and plated on LB agar to determine colony-forming units (CFU). A nonlinear model was used to fit the experimental data to a biphasic kill curve (see Materials and Methods). Number of biological replicates, n = 4. Data at each time point is presented as mean ± standard error (SEM).

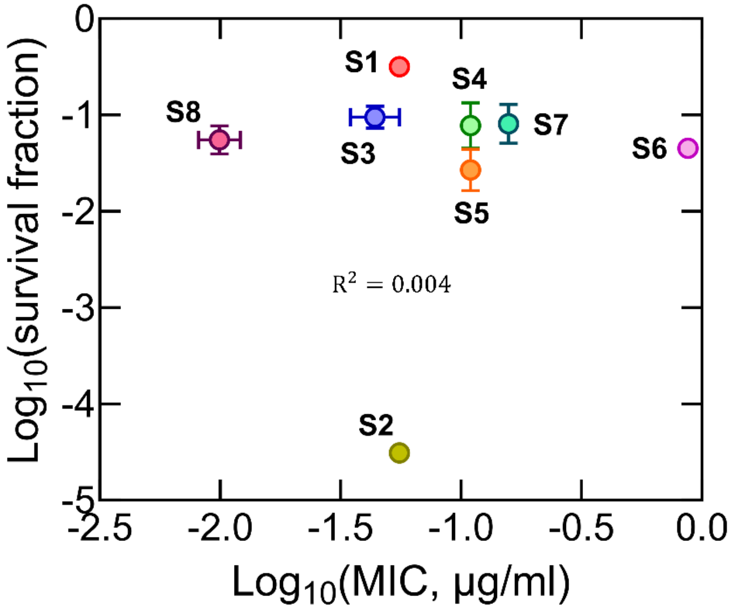

Fig. S2. Correlation between survival fraction and MIC of mutant and parental strains (WT). The survival fraction and MIC of the mutant and WT strains were plotted, and Pearson Correlation was used for the correlation analysis. MICs for samples S1–S8 were determined using ofloxacin ETEST strips. n=3. Data corresponding to each time point represents mean value ± standard error (SEM).

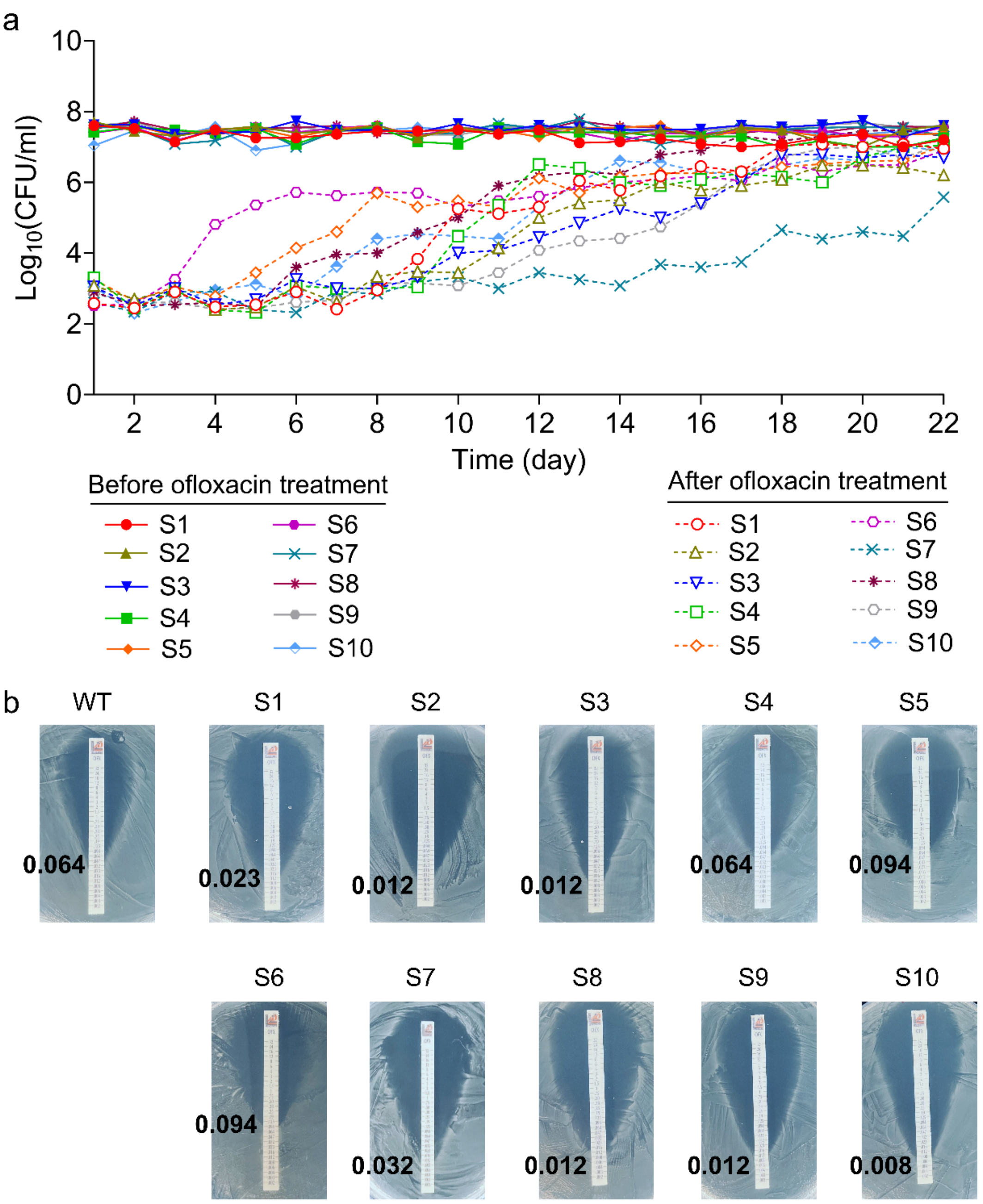

Fig. S3. Generation of ofloxacin mutant strains using adaptive laboratory evolutionary experiment. (a) Stationary-phase *E. coli* MG1655 MO cells (16 h culture) were diluted 1:100 in 2 mL LB in test tubes and treated with ofloxacin (5 µg/ml) for 7 h. After washing, half of the cells were transferred to fresh LB for overnight recovery, and the remaining half was plated to determine the CFU levels. This cycle was repeated daily for 22 days. CFU levels before and after treatment are shown in the plot. n = 1. **(b)** Stationary-phase *E. coli* MG1655 MO cells and evolved strains

were diluted in LB medium to a final density of ~10^8 CFU/ml and spread on agar plates. ETEST strips were applied to determine the minimum inhibitory concentration (MIC), followed by incubation at 37 °C for at least 16 h. MIC values were determined at the point where the bacterial growth inhibition ellipse intersected the antibiotic strip, with the corresponding concentrations indicated in the figure. The antibiotic concentration unit on strips is µg/ml. A representative biological replicate is shown, with all three biological replicates consistently yielding similar trends. n = 3.

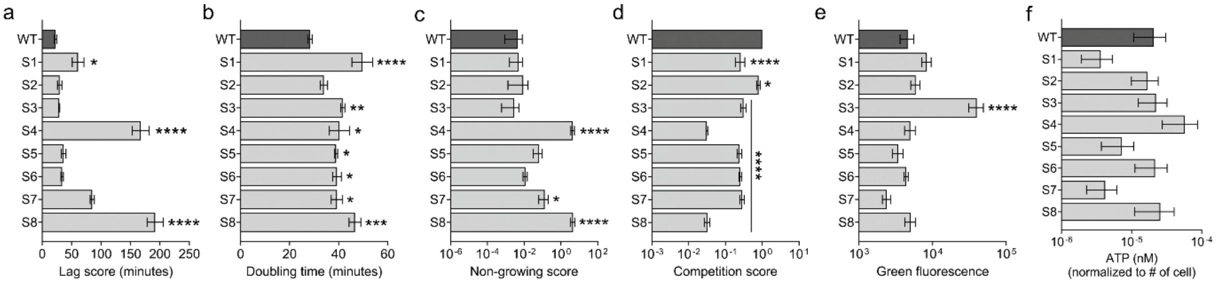

Fig. S4. Determination of fitness factors and metabolic activities of mutant strains. (a) Lag scores of the indicated strains were calculated using the growth curves (**Fig. 2a** and Materials and Methods). n=4. **(b)** The doubling times of the indicated strains were calculated using data from the exponential growth phase of their growth curves (**Fig. 2a** and Materials and Methods). n=4. **(c)** The protein dilution method was used to quantify non-growing cells (**Fig. 2b**). The ratio of non-growing to growing cell levels was defined as the non-growing score. n=4. **(d)** The parental and mutant cells were cocultured in LB. At t=24, cells were collected, diluted in PBS and analyzed with a flow cytometer at single cell levels (**Fig. 2c**). The ratio between the levels of mutants' cells to WT strain is defined as the competition score. n=4. **(e)** Stationary phase cells of indicated strains were stained with RSG dye and analyzed with a flow cytometer to determine the metabolic state of the cells. RSG dye emits green fluorescence when it gets reduced by the bacterial reductases which indirectly shows the metabolic state of the cells. n=4. **(f)** Stationary phase cells of the indicated strains were collected to measure the intracellular ATP concentrations (see Materials and Methods). A flow cytometer was used to count cell numbers for normalization purposes. n=4. Statistical analysis was performed between the WT and single mutants using one-way ANOVA with Dunnett's post-test. *P<0.05, **P < 0.01, ***P < 0.001, and ****P < 0.0001. Data corresponding to each time point represents mean value ± standard error (SEM).

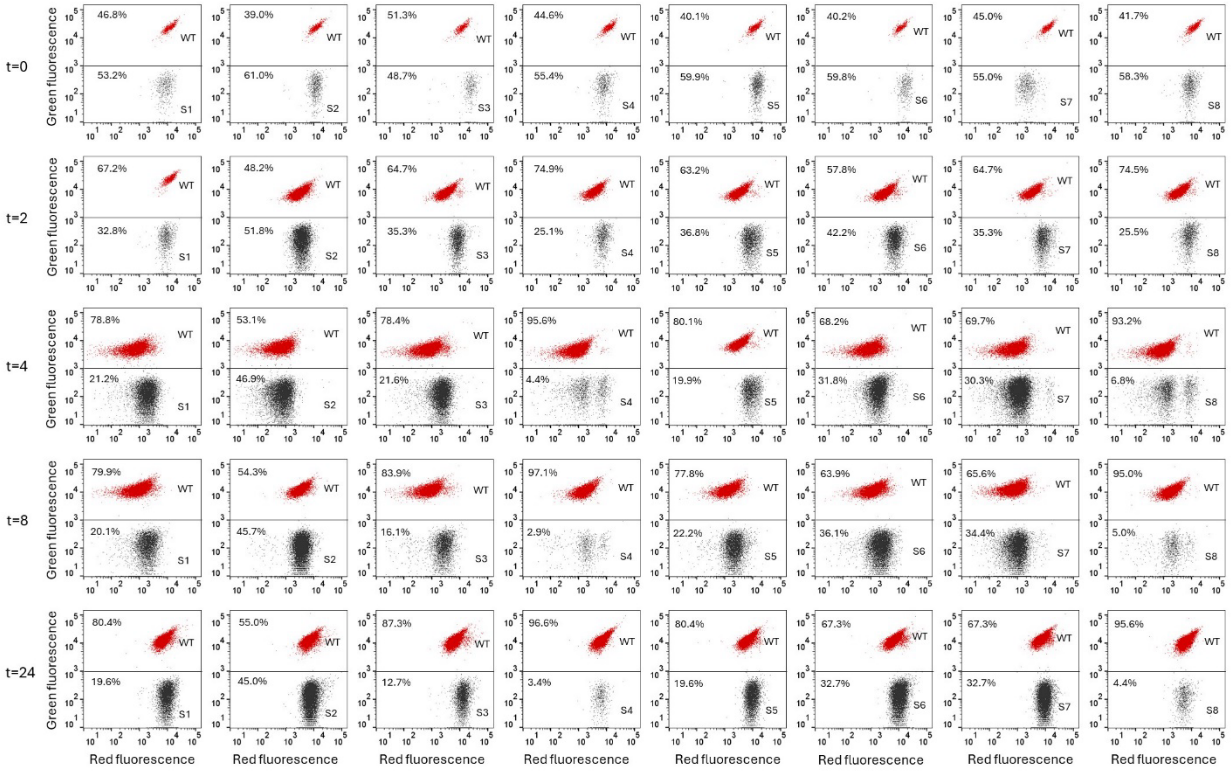

Fig. S5. Monitoring the time-dependent growth dynamics of mutant and parental WT strains. Evolved mutants (harboring pUA66-EV) and WT cells (harboring pUA66-*gfp*) were cultured individually overnight (16 h) in the presence of 1 mM IPTG in LB media. Stationary phase cells of the mutant and wild-type strains were diluted in LB to achieve an equal number of cells for each strain and co-cultured in the presence of 1 mM IPTG for 24 h. At designated time points, cells were collected, diluted in PBS and analyzed with a flow cytometer at the single-cell level. A representative biological replicate is shown, with all three biological replicates consistently yielding similar trends. n=3.

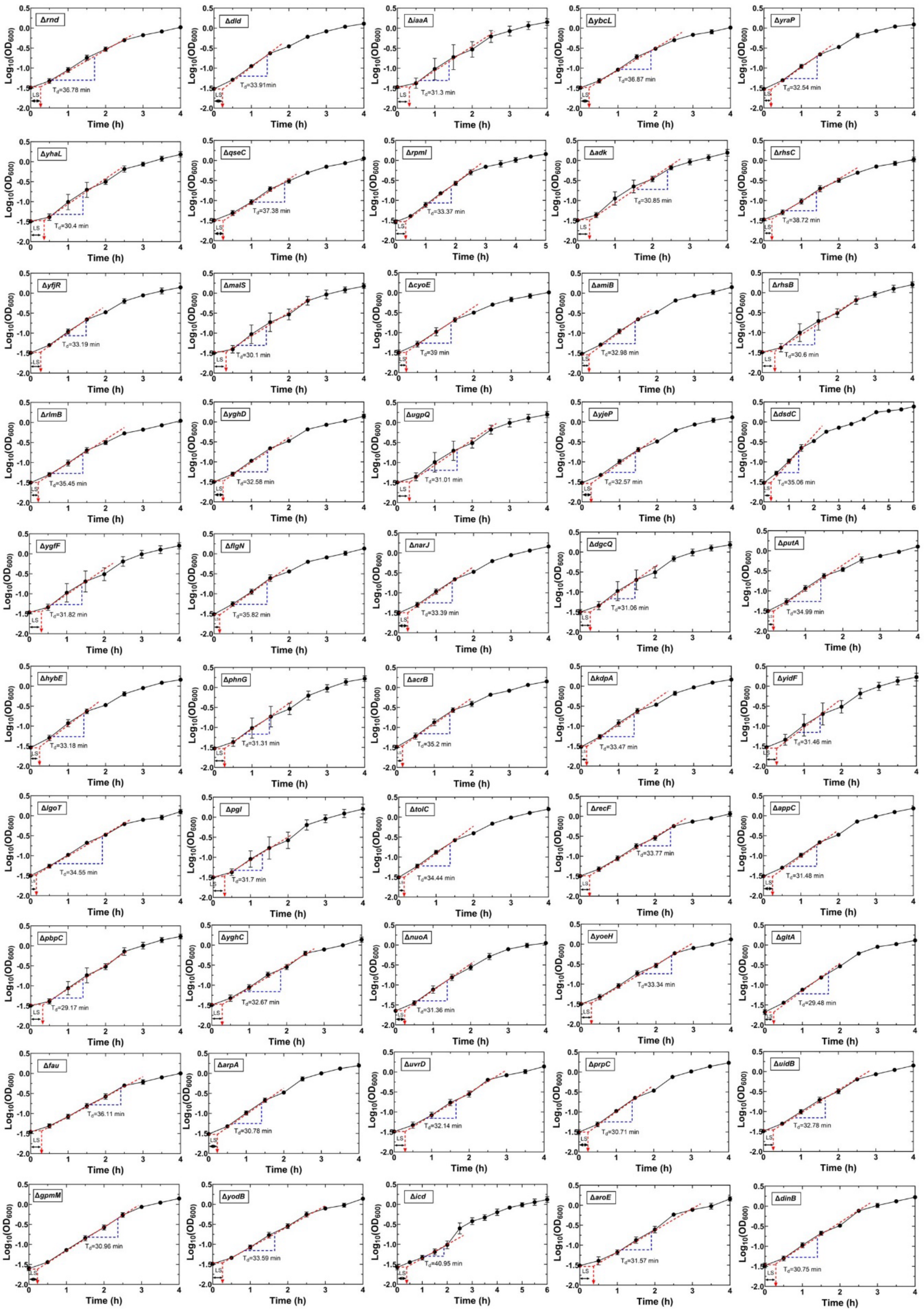
Log10(OD600)
Time (h)
LS
ΔrndTd=36.78 min
ΔdldTd=33.91min
ΔiaaATd=31.3 min
ΔybcLTd=36.87 min
ΔyraPTd=32.54 min
ΔyhaLTd=30.4 min
ΔqseCTd=37.38 min
ΔrpmITd=33.37 min
ΔadkTd=30.85 min
ΔrhsCTd=38.72 min
ΔyfjRTd=33.19 min
ΔmalSTd=30.1 min
ΔcyoETd=39 min
ΔamiBTd=32.98 min
ΔrhsBTd=30.6 min
ΔrlmBTd=35.45 min
ΔyghDTd=32.58 min
ΔugpQTd=31.01 min
ΔyjePTd=32.57 min
ΔdsdCTd=35.06 min
ΔygfFTd=31.82 min
ΔflgNTd=35.82 min
ΔnarJTd=33.39 min
ΔdgcQTd=31.06 min
ΔputATd=34.99 min
ΔhybETd=33.18 min
ΔphnGTd=31.31 min
ΔacrBTd=35.2 min
ΔkdpATd=33.47 min
ΔyidFTd=31.46 min
ΔlgoTTd=34.55 min
ΔpglTd=31.7 min
ΔtolCTd=34.44 min
ΔrecFTd=33.77 min
ΔappCTd=31.48 min
ΔpbpCTd=29.17 min
ΔyghCTd=32.67 min
ΔnuoATd=31.36 min
ΔyoeHTd=33.34 min
ΔgltATd=29.48 min
ΔfauTd=36.11 min
ΔarpATd=30.78 min
ΔuvrDTd=32.14 min
ΔprpCTd=30.71 min
ΔuidBTd=32.78 min
ΔgpmMTd=30.96 min
ΔyodBTd=33.59 min
ΔicdTd=40.95 min
ΔaroETd=31.57 min
ΔdinBTd=30.75 min

Fig. S6. Growth curves represent lag scores and doubling times of knockout strains. Stationary phase cells were diluted 100-fold in LB media and cultured for 24 h. At designated time points, cells were collected to measure OD_{600} using a plate reader. These growth curves were utilized to determine the lag and doubling time scores (for details see Materials and Methods). n=4. Data corresponding to each time point represents mean value ± standard error (SEM).

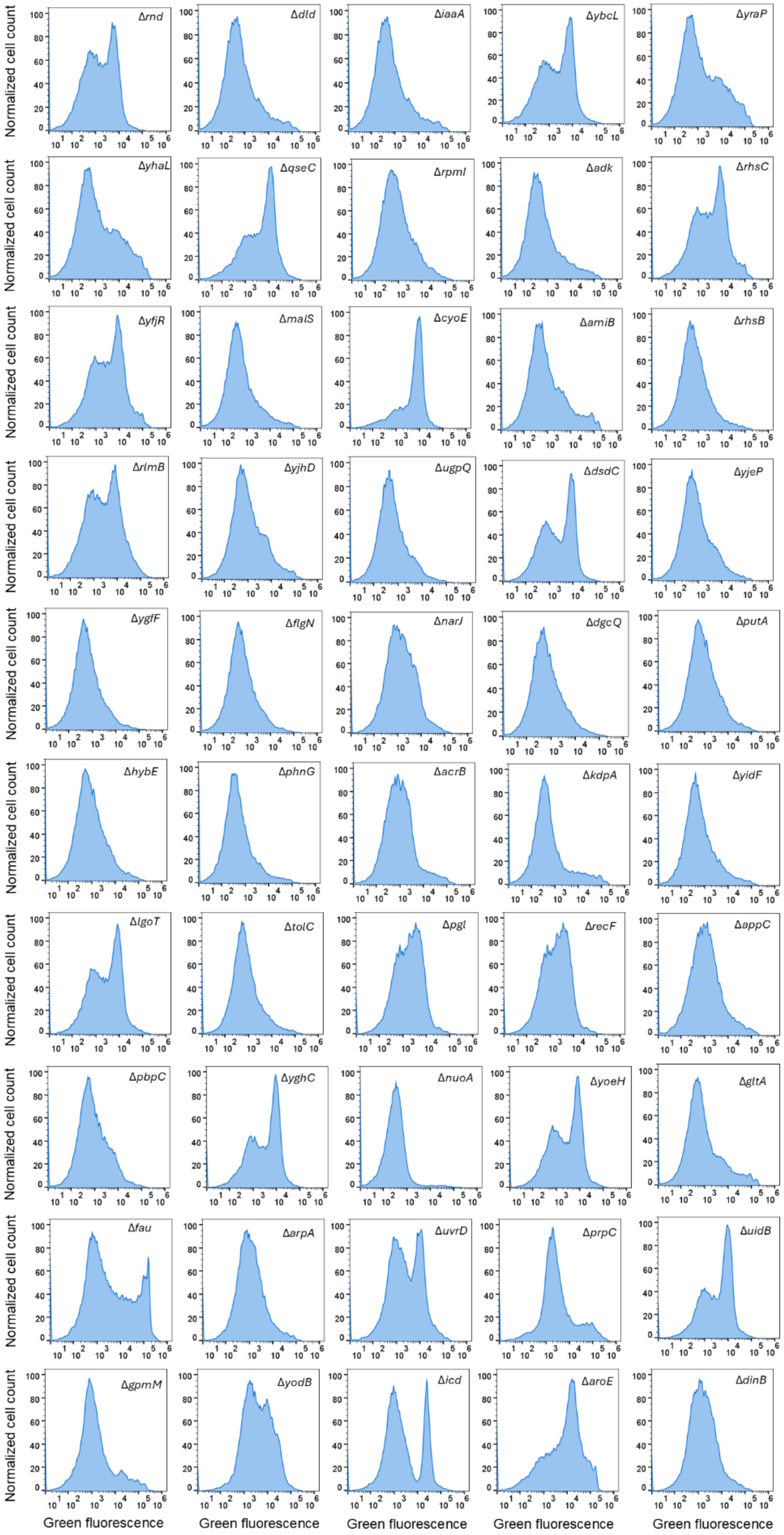
Normalized cell count
Green fluorescence
Δrnd
Δdld
ΔiaaA
ΔybcL
ΔyraP
ΔyhaL
ΔqseC
ΔrpmI
Δadk
ΔrhsC
ΔyfjR
ΔmalS
ΔcyoE
ΔamiB
ΔrhsB
ΔrlmB
ΔyjhD
ΔugpQ
ΔdsdC
ΔyjeP
ΔygfF
ΔflgN
ΔnarJ
ΔdgcQ
ΔputA
ΔhybE
ΔphnG
ΔacrB
ΔkdpA
ΔyidF
ΔlgoT
ΔtolC
Δpgl
ΔrecF
ΔappC
ΔpbpC
ΔyghC
ΔnuoA
ΔyoeH
ΔgltA
Δfau
ΔarpA
ΔuvrD
ΔprpC
ΔuidB
ΔgpmM
ΔyodB
Δicd
ΔaroE
ΔdinB

Fig. S7. RSG staining of knockout strains. Stationary phase cells of indicated knockout strains were stained with Redox Sensor Green (RSG) dye and analyzed with a flow cytometer to measure their metabolic activities. A representative flow cytometry diagram is shown. All independent biological replicates showed a similar trend. n = 4.

Table S1: Bacterial strains and plasmids used in this study.

Bacterial strains	Source
Escherichia coli K-12 MG1655 Wild Type (WT)	Gift from Dr. Mark P. Brynildsen
Escherichia coli K-12 MG1655 MO Cured	Gift from Dr. Mark P. Brynildsen
Evolved mutant S1	This study
Evolved mutant S2	This study
Evolved mutant S3	This study
Evolved mutant S4	This study
Evolved mutant S5	This study
Evolved mutant S6	This study
Evolved mutant S7	This study
Evolved mutant S8	This study
Escherichia coli K-12 MG1655 Δ*icd*	This study
Escherichia coli K-12 MG1655 Δ*cyoE*	This study
Escherichia coli K-12 MG1655 Δ*lgoT*	This study
Escherichia coli K-12 MG1655 Δ*yghC*	This study
Escherichia coli K-12 MG1655 Δ*tolC*	This study
Escherichia coli K-12 MG1655 Δ*rnd*	This study
Escherichia coli K-12 MG1655 Δ*dld*	This study
Escherichia coli K-12 MG1655 Δ*acrB*	This study
Escherichia coli K-12 MG1655 Δ*ybcL*	This study
Escherichia coli K-12 MG1655 Δ*uidB*	This study
Escherichia coli K-12 MG1655 Δ*dsdC*	This study
Escherichia coli K-12 MG1655 Δ*narJ*	This study
Escherichia coli K-12 MG1655 Δ*yoeH*	This study
Escherichia coli K-12 MG1655 Δ*recF*	This study
Escherichia coli K-12 MG1655 Δ*rhsC*	This study
Escherichia coli K-12 MG1655 Δ*dinB*	This study
Escherichia coli K-12 MG1655 Δ*flgN*	This study
Escherichia coli K-12 MG1655 Δ*hybE*	This study

Escherichia coli K-12 MG1655 Δ*yjeP*	This study
Escherichia coli K-12 MG1655 Δ*yghD*	This study
Escherichia coli K-12 MG1655 Δ*yfjR*	This study
Escherichia coli K-12 MG1655 Δ*pgl*	This study
Escherichia coli K-12 MG1655 Δ*putA*	This study
Escherichia coli K-12 MG1655 Δ*amiB*	This study
Escherichia coli K-12 MG1655 Δ*rlmB*	This study
Escherichia coli K-12 MG1655 Δ*yodB*	This study
Escherichia coli K-12 MG1655 Δ*kdpA*	This study
Escherichia coli K-12 MG1655 Δ*appC*	This study
Escherichia coli K-12 MG1655 Δ*arpA*	This study
Escherichia coli K-12 MG1655 Δ*pbpC*	This study
Escherichia coli K-12 MG1655 Δ*rpmI*	This study
Escherichia coli K-12 MG1655 Δ*prpC*	This study
Escherichia coli K-12 MG1655 Δ*yraP*	This study
Escherichia coli K-12 MG1655 Δ*qseC*	This study
Escherichia coli K-12 MG1655 Δ*yhaL*	This study
Escherichia coli K-12 MG1655 Δ*iaaA*	This study
Escherichia coli K-12 MG1655 Δ*malS*	This study
Escherichia coli K-12 MG1655 Δ*aroE*	This study
Escherichia coli K-12 MG1655 Δ*ugpQ*	This study
Escherichia coli K-12 MG1655 Δ*rhsB*	This study
Escherichia coli K-12 MG1655 Δ*yidF*	This study
Escherichia coli K-12 MG1655 Δ*phnG*	This study
Escherichia coli K-12 MG1655 Δ*dgcQ*	This study
Escherichia coli K-12 MG1655 Δ*ygfF*	This study
Escherichia coli K-12 MG1655 Δ*adk*	This study
Escherichia coli K-12 MG1655 Δ*gpmM*	This study
Escherichia coli K-12 MG1655 Δ*gltA*	This study
Escherichia coli K-12 MG1655 Δ*fau*	This study

Escherichia coli K-12 MG1655 Δ*nuoA*	This study
Escherichia coli K-12 MG1655 Δ*uvrD*	This study
**Plasmids**	**Source**
pUA66-empty vector (EV)	Gift from Dr. Mark P. Brynildsen
pUA66-*gfp*	Gift from Dr. Mark P. Brynildsen

Table S2: The sequencing data revealed three distinct types of mutations: insertions and deletions (INDELs), structural variations (SVs), and single nucleotide polymorphisms (SNPs).

**The INDEL mutations of the evolved strains**							
**S1**	**S2**	**S3**	**S4**	**S5**	**S6**	**S7**	**S8**
carB	*dinB*	*ybiB*	*yccE*	*cyoE*	*yebT*	*panE*	*thrS*
cyoE	*nagE*	*topA*	*narJ*	*ybcL*	*mqo*	*ybaZ*	*recJ*
asr	*hrpA*	*gadC*	*metG*	*yafS*	*recD*	*igaA*	*tolC*
dgcQ	*yfjR*	*flu*	*yfaS*	*yfcP*	*ugpQ*	*lldD*	*gyrB*
valU	*rpoD*	*yghB*	*intA*	*qseC*	*hdfR*	*fdoG*	*hemX*
argQ	*yheT*	*ftsX*	*yfjM*	*rlmB*	*metH*		
ttdT		*wecC*	*ygiD*				
yhbQ			*ugpQ*				
rpoA							
yicH							
yjaG							
phnM							
aspA							
ytfE							
**The SV mutations of the evolved strains**							
**S1**	**S2**	**S3**	**S4**	**S5**	**S6**	**S7**	**S8**
rhsC	*rhsC*	*insL*	*dinB*	*mhpT*	*rhsC*	*prpC*	*yeeS*
uidB	*iaaA*	*gltA*	*yagF*	*mhpT*	*yehH*	*mphF*	*metG*

rpnB	*narJ*	*pgl*	*fepB*	*cusS*	*lepB*	*astB*	*fau*
hypF	*insA*	*serS*	*flgN*	*tus*	*hypC*	*ypfG*	*hybE*
pitB	*ygcR*	*appC*	*ymfH*	*yoeH*	*rhsB*	*adk*	*patA*
infB	*ugpQ*	*putA*	*rspR*	*yghD*	*malS*	*recF*	*yhaL*
rhsB	*malS*	*nuoA*	*astD*	*soxR*			*zntA*
yidF	*rhsA*	*dsdC*	*pbpC*	*phnG*			*uvrD*
phnG	*gpmM*	*ppdC*	*ygfF*	*yaiL*			*hybF*
rlmB	*yifE*	*bcsB*	*argK*	*fumC*			
	tamA	*basS*	*yghC*	*yaiL*			
	tamB	*basR*	*phnI*				
	lptF	*dmsA*					
	tsr	*hyaF*					
	ygcQ						
	ygcS						
	avtA						
	lptG						
	yifB						
	hdfR						
	lgoT						
**The SNP mutations of the evolved strains**							
**S1**	**S2**	**S3**	**S4**	**S5**	**S6**	**S7**	**S8**
dinB	*rutG*	*kdpA*	*metG*	*gpmM*	*adk*	*panE*	*acrB*
poxB	*icd*	*icd*	*parE*	*icd*	*ybhD*	*adk*	*clpA*
icd	*yfjR*	*topA*	*gyrB*	*hybA*	*gsiD*	*icd*	*icd*
topA		*rnd*	*nrdD*	*pitA*	*speG*	*hyuA*	*ynjF*
bglH		*hcaD*		*uhpC*	*atoC*	*glpD*	*metG*
rpoB		*infB*		*arpA*	*mreB*	*aslB*	*tolC*
						amiB	*gyrB*
							yjiJ

Table S3: Oligonucleotides for the generation of the knockout strains.

Mutations	Forward Primer (5' to 3')	Reverse Primer (5' to 3')	Source
Δ*icd*::KAN^R	TGGCAGACGAGCAAACC AGTAGCGCTCGAAGGAG AGGTGAGTGTAGGCTGG AGCTGCTTC	ACAACGGGAGCGTTACG CTCCCGTTAATAAATTT AACAAACTACGGCATTA ACGGCTGACATGGGAAT	IDT DNA Inc.
Δ*cyoE*::KAN^R	ACCTCAACTACAACATG ATGATGCACTAAGAGCG GCGGTTGTGTAGGCTGG AGCTGCTTC	AAAAAAGGTGCCGTAG CACCTTTTTAATAGAGA GGTTTTGTTAACGGCTG ACATGGGAAT	IDT DNA Inc.
Δ*lgoT*::KAN^R	ATTACGTAAACAATTAA AATTATAAATACAGGAG TCCGGCGTGTAGGCTGG AGCTGCTTC	AAAATGCCCGATAAGCA AAATGTTATCGGGCATA AGGAGATTAACGGCTGA CATGGGAAT	IDT DNA Inc.
Δ*yghC*::KAN^R	ATTGAAATTGTAGAACA AGCCCTTGAAAAGGAAT AACAAAGTGTAGGCTGG AGCTGCTTC	GGGTTATGGTTTGACTC ATTGTTTATCTCCTCGTT TTCGCTTAACGGCTGAC ATGGGAAT	IDT DNA Inc.
Δ*tolC*::KAN^R	TTTGATCGCGCTAAATAC TGCTTCACCACAAGGAA TGCAAGTGTAGGCTGGA GCTGCTTC	GTTGCCTTACGTTCAGA CGGGGCCGAAGCCCCGT CGTCGTCATTAACGGCT GACATGGGAAT	IDT DNA Inc.
Δ*rnd*::KAN^R	ACGCCGGTTAATCCGGC GTTTTTTTTGACGCCCAC TAAAGAGAAAACAATGT GTAGGCTGGAGCTGCTTC	GATGTGGCGCTGAGCGC GCCCAGTCCGGCTTCGG AAGATTTTAACGGCTGA CATGGGAAT	IDT DNA Inc.
Δ*dld*::KAN^R	CTAGTTTGTGATATTTTT TCGCCACCACAAGGAGT GGAAAGTGTAGGCTGGA GCTGCTTC	TAAGTGAATTCGGATGG CGATACTCTGCCATCCG TAATTTTTAACGGCTGA CATGGGAAT	IDT DNA Inc.

Δ*acrB*::KAN^R	GAACAGTCCAAGTCTTAACTTAAACAGGAGCCGTTAAGACGTGTAGGCTGGAGCTGCTTC	AAAAAGGCCGCTTACGCGGCCTTAGTGATTACACGTTGTATTAACGGCTGACATGGGAAT	IDT DNA Inc.
Δ*ybcL*::KAN^R	ATCAAATAAAATAACGATAGGTCATAACAAAGAGGTTTTTGTGTAGGCTGGAGCTGCTTC	TCTTTAAGTTAGTCTTACCTCTTGCCAGCATACTCTCACCTTAACGGCTGACATGGGAAT	IDT DNA Inc.
Δ*uidB*::KAN^R	GCATGAACTTCGGTGAAAAACCGCAGCAGGGAGGCAAACAGTGTAGGCTGGAGCTGCTTC	AGAGGCAGCCGTCAGGCAAATAACGGCCATGGCCACTATTTTTCTCATAGTTGCACCTTTGATGTTCTGATTATTTTTATTGAATATTAACGGCTGACATGGGAAT	IDT DNA Inc.
Δ*dsdC*::KAN^R	GCCGCTATTTTTTACACTTAAGCGAAAAATGAGGTGATTGTGTAGGCTGGAGCTGCTTC	AGGCCGAATAAGGCACACCAGGAACTCGTGGTTTTATTCATTAACGGCTGACATGGGAAT	IDT DNA Inc.
Δ*narJ*::KAN^R	TCGATGCCATCGATGTGACCAGCAAAACGGAGCCGCATCCGTGTAGGCTGGAGCTGCTTC	GAAGACCGCCCCGGCTATGTACGGGTAGATATCAAAGAAGAACATATTCAGGAATTGCATTAACGGCTGACATGGGAAT	IDT DNA Inc.
Δ*yoeH*::KAN^R	CTGACTCGTGTCAGTCGGTCTCTGGTTGTATCCACGGACCGTGTAGGCTGGAGCTGCTTC	GTCATCACTCTGGCCATTAGAGTAACCTTCTGCATTCATCCTTTTGTAAAAAGTTTATATTAGTATCAGCAATTAACCGGATTAACGGCTGACATGGGAAT	IDT DNA Inc.

Δ*recF*::KAN^R	GCCAGAGCGCGGCTTAT GTTGTCATGCCAATGAG ACTGTAGTGTAGGCTGG AGCTGCTTC	AGAATTCGACATCAACG TTTCTCGCTCATTTATAC TTGGGTTAACGGCTGAC ATGGGAAT	IDT DNA Inc.
Δ*rhsC*::KAN^R	TGTCAGCAGACAAATAA CCCGATAAAACAAGGAT GAGCAGGTGTAGGCTGG AGCTGCTTC	GCATGGAAAAGAATAG TATTAAAAGCGATAAAG CCAGCCATATAGCTCTG AATAAAAAAATAATATA TTTCATTAACGGCTGAC ATGGGAAT	IDT DNA Inc.
Δ*dinB*::KAN^R	TGAAATCACTGTATACTT TACCAGTGTTGAGAGGT GAGCAGTGTAGGCTGGA GCTGCTTC	GCATACAGTGATACCCT CATAATAATGCACACCA GAATATACATAATAGTA TACATTAACGGCTGACA TGGGAAT	IDT DNA Inc.
Δ*flgN*::KAN^R	ATCAACGAAGCGCAGCA AGACTTGCAGAGTAACT GATCGTGTGTAGGCTGG AGCTGCTTC	ACAATGCATTCCGGCCT GCAGTGCAGGCCGGAG ATAATCTTTAACGGCTG ACATGGGAAT	IDT DNA Inc.
Δ*hybE*::KAN^R	CGTGAATCTGGCGTGGA AGCCATCCCACGGGAGG CGATTCGTGTAGGCTGG AGCTGCTTC	GCCGTTGGATAATTTCA ACGGCGCTCTGGCAAAG AGACAACTTAACGGCTG ACATGGGAAT	IDT DNA Inc.
Δ*yjeP*::KAN^R	CAAAAAAGACCAGGTCT AATCCATCAAAGGAAAC GCTGACGTGTAGGCTGG AGCTGCTTC	CAGTTTTGTTTGTGAGC CGGATTGGTTCATCCGG CACACAAACTTAACGGC TGACATGGGAAT	IDT DNA Inc.
Δ*yghD*::KAN^R	GATGCGGTTTCCGGCGG CGTAACGTTGCGGAGGA AATAACGTGTAGGCTGG AGCTGCTTC	CTGGGGGAATTACTCTC GCCACGTTAACGAGAGT AATTTTATTGATATTAA	IDT DNA Inc.

		TCTCCTGATACTTTAAC GGCTGACATGGGAAT	
Δ*yfjR*::KAN^R	CCCCCCTCTTTAAAGTAA TCACATCATTTTCAGTCA GTTAACTTTCCTGGAGAA TCTCTCGTGTAGGCTGGA GCTGCTTC	TGGGCTCCTCCATGTTG CCGGGTTGATAACAACG CCTGCGTTAACGGCTGA CATGGGAAT	IDT DNA Inc.
Δ*pgl*::KAN^R	TTAGCTGTTACAGTCAGT TGCTAAATGCAAAGGAG CATTCGTGTAGGCTGGA GCTGCTTC	TACACCGGCGCAGGAG AGACTGCGCCGGGTAAA TCAGCGGTTAACGGCTG ACATGGGAAT	IDT DNA Inc.
Δ*putA*::KAN^R	AACGTTAAGTTGCACCTT TCTGAACAACAGGAGTA ATGGCGTGTAGGCTGGA GCTGCTTC	AAGATGCCGGAGGAGG TTGTAACATCCTCCGGC TACCTGTTTAACGGCTG ACATGGGAAT	IDT DNA Inc.
Δ*amiB*::KAN^R	GCTGGCGCGTTTAGCCG GTTAACCTTTGAAAGGTG GCGGGGTGTAGGCTGGA GCTGCTTC	CCAGTTGTGGCGGTAAG ACCTGAATTGGCATCAA TCGTCCTTAACGGCTGA CATGGGAAT	IDT DNA Inc.
Δ*rlmB*::KAN^R	ACGTAGTGCATCAGGCA AAACGTAAACAACGAGT ACATTAGTGTAGGCTGG AGCTGCTTC	AACCATCCAAATCTGGA TGGCTTTTCATAATTCTG AGAAATTAACGGCTGAC ATGGGAAT	IDT DNA Inc.
Δ*yodB*::KAN^R	GTGGGATAGCTTGACTGT GAAAATCACAGGAGCTA CAAAAGTGTAGGCTGGA GCTGCTTC	GACGCGTTGATGTCCAT GGTTCACATTTCCTTGT AAGGGGATCGACTTAAC GGCTGACATGGGAAT	IDT DNA Inc.
Δ*kdpA*::KAN^R	GTTATCTGGTTTATGCCC TGATCAATGCGGAGGCG TTCTGGTGTAGGCTGGAG CTGCTTC	GCGCCAGTTGTTTACGA CTCATATTCAGTGCTCA CTCAATATCATTAACGG CTGACATGGGAAT	IDT DNA Inc.

Δ*appC*::KAN^R	TTATCGGGTTATTTTTCT CTCTTCGCCTACAGGAGT GCGCGTGTAGGCTGGAG CTGCTTC	ATGAAGCGCAATGTTTC ATAATCAAACATGATTT TCTCCTTTAACGGCTGA CATGGGAAT	IDT DNA Inc.
Δ*arpA*::KAN^R	CACTGTTCTGATAGTTAA AATTCAAGACATCAATA AACAATGAGATATTTAA GTGTAGGCTGGAGCTGC TTC	CATGTGGAAGATGTTTA TGCGTATCGGCGCAGGC AAAGATTTAACGGCTGA CATGGGAAT	IDT DNA Inc.
Δ*pbpC*::KAN^R	GACCGGCGCGGCTGAAG ATCTGCTGATTGTCAGAC CGTAAGTGTAGGCTGGA GCTGCTTC	GGGCCGTTGACTATTGA TGAAGGTAACATTTTTT TAAAATAAAATGAGTTT TAGCAACAATCAGGGAC AAAACAGATTAACGGCT GACATGGGAAT	IDT DNA Inc.
Δ*rpmI*::KAN^R	TTGTTTCGTTTATTAACA ATGCGAAGTGGAAGTTA TTAAAGTGTAGGCTGGA GCTGCTTC	GTATCTATATTCTAATT AAAAAGTTAAAAACGTT AACGGCTTAACGGCTGA CATGGGAAT	IDT DNA Inc.
Δ*prpC*::KAN^R	CAATCTCGACCCTACAA ATGATAACAATGACGAG GACAACGTGTAGGCTGG AGCTGCTTC	CTGACATTGGGTACGTT TCCTTATTGTTATTCGTA GAGGT TTAACGGCTGACATGGG AAT	IDT DNA Inc.
Δ*yraP*::KAN^R	TAACACGCTTTTCCCTCA CCAGGATGATTAAGGAG AATACGTGTAGGCTGGA GCTGCTTC	AAGACGCGGCAAGCGT CGCATCAGGCATTACAA GGGGCTGTTAACGGCTG ACATGGGAAT	IDT DNA Inc.
Δ*qseC*::KAN^R	TTCGTACCGTGCATGGTA TTGGTTACACATTAGGTG	TCTATGAGAGTCGTTTT AACGGCTCTCATAGACA	IDT DNA Inc.

	AGAAGTGTAGGCTGGAG CTGCTTC	GAGAAGTTAACGGCTGA CATGGGAAT	
Δ*yhaL*::KAN^R	CGCTTTGCTGATAGATTT GCCTGTCTAGTTGTTTTT ACAGGGAGATGATGGTG TAGGCTGGAGCTGCTTC	GGCCGGATAAGGCGTTT TCGCCGCATCCGGCAAC CTGTCTTTAACGGCTGA CATGGGAAT	IDT DNA Inc.
Δ*iaaA*::KAN^R	TATAGCAAAAGTGGCGA ACCACCCTTAATGGACG AATACTGTGTAGGCTGG AGCTGCTTC	CAGATTTTCAACCGCCA GCACATTACCGGCATCA AGTTCATTAACGGCTGA CATGGGAAT	IDT DNA Inc.
Δ*malS*::KAN^R	GCAAACTGTCTGAAATC GCAGCAATAAGGACTCA TCCGCCGTGTAGGCTGG AGCTGCTTC	GCGGCTTCCCGAGGTAC TGACGAACGGGAAGCC GGAAAAGTTAACGGCTG ACATGGGAAT	IDT DNA Inc.
Δ*aroE*::KAN^R	GATGCCCTGACGGGTGA ACTGTTTCGACAGGGGT AACATAGTGTAGGCTGG AGCTGCTTC	TCGTCCCACTCTTCCCTG TCCGGAAACTGGATGGC CTGATTTAACGGCTGAC ATGGGAAT	IDT DNA Inc.
Δ*ugpQ*::KAN^R	ATCAGCTGCATCTTTTTG ATGGTGAAACAGGACAA CGAGTGTGTAGGCTGGA GCTGCTTC	GAATCAGCCCAGCACGC CGCAGCCTGACATTCCG TTGAAAATTAACGGCTG ACATGGGAAT	IDT DNA Inc.
Δ*rhsB*::KAN^R	TGAAGATACTGTCATTAA AATAATAGAAAAGGATT TTACGGTGTAGGCTGGA GCTGCTTC	CAAACAAAGATACAAT GACACAGTTCCTAAACA ATATTTTTTTAACGGCT GACATGGGAAT	IDT DNA Inc.
Δ*yidF*::KAN^R	CGCAATACTG TTTGCTGTAA CGCATATACA TCAACTTATC	ATGTCGCATCAGGGGCA GCCCGTTTAAGCGCACG TTAACGGCTGACATGGG AAT	IDT DNA Inc.

	GTATTTATTG AGAGAGTCGCGTGTAGG CTGGAGCTGCTTC		
Δ*phnG*::KAN^R	GACGCGCGCCGACATGA TTGAATTCACTATGGAGC ACTGAGTGTAGGCTGGA GCTGCTTC	TGGGCATCCTGCACGGG AAGCATAAAAGCGGTTT CCAGGGTTAACGGCTGA CATGGGAAT	IDT DNA Inc.
Δ*dgcQ*::KAN^R	TATGCCAGAATCATAAA AAAGCAGGTTGGGAGTC GTCAGGGTGTAGGCTGG AGCTGCTTC	AAGGATGGCGTGAAGG GCTGGACCATTTTTCTC CGCCCGTTAACGGCTGA CATGGGAAT	IDT DNA Inc.
Δ*ygfF*::KAN^R	CAAAGATACGTCAACGA ATTAATTTTTCTCGGAAA AACAAGTGTAGGCTGGA GCTGCTTC	GGATGCAATACTTGTTG CATCCGGTCAGACAACT TCCCTGTTTTAACGGCT GACATGGGAAT	IDT DNA Inc.
Δ*adk*::KAN^R	CAATCGCCTGTTGGTGGT ATCGTTTATCGCTTTTTC AAAAAATTCGACACATT TTAAGGGGATTTTCGCAG TGTAGGCTGGAGCTGCTT C	CCCCGAGGGGGCGAGG GGACTGTCCGTGCGCGC TTTCGAATTAACGGCTG ACATGGGAAT	IDT DNA Inc.
Δ*gpmM*::KAN^R	AATTACGCAAATTTTGAC TCTTGAGTATGAGGTTGT CGCAGTGTAGGCTGGAG CTGCTTC	GTGTCATGGTATTAATC GCCTTTCCCCTCATGGG GAGGGATTAACGGCTGA CATGGGAAT	IDT DNA Inc.
Δ*gltA*::KAN^R	TCCGGCAGTCTTACGCAA TAAGGCGCTAAGGAGAC CTTAAGTGTAGGCTGGA GCTGCTTC	GAACGGCGGGTTAAAAT ATTTACAACTTAGCAAT CAACCATTAACGGCTGA CATGGGAAT	IDT DNA Inc.
Δ*fau*::KAN^R	AGAACTCCCACTGACATT ATCCCGACAAGAAATCC	GAATCAACGATGTCAAT CAGGGCGATGCGGGTGT	IDT DNA Inc.

	GCAAAGTGTAGGCTGGAGCTGCTTC	ATCGCCCTTAACGGCTGACATGGGAAT	
Δ*nuoA*::KAN^R	GAAGAGCAGTGAATCTGGCGCTACTTTTGATGAGTAAGCAGTGTAGGCTGGAGCTGCTTC	CTATGCGGGTGAGCGTATAATCCATCTTAATGCCTCGCGGTTAACGGCTGACATGGGAAT	IDT DNA Inc.
Δ*uvrD*::KAN^R	TGCGCTTCTCCGCCCAACCTATTTTTACGCGGCGGTGCCAGTGTAGGCTGGAGCTGCTTC	TTTAGGCCAAATAAGGTGCGCAGCACCGCATCCGGCAACGTTAACGGCTGACATGGGAAT	IDT DNA Inc.

Table S4: Oligonucleotides for the verification of the knockout strains.

Oligonucleotides to verify gene deletions					
**Mutations**	**External forward primer (5' to 3')**	**External reverse primer (5' to 3')**	**Internal forward primer (5' to 3')**	**Internal reverse primer (5' to 3')**	**Source**
Δ*icd*::KAN^R	CATGACGGCAAACAATAGGGTAG	GCTCTTCGTCCAGATCATCCT	GGCACAAGGCAAGAAGATCAC	GTGGGTGGCTTCAAACAGG	IDT DNA Inc.
Δ*cyoE*::KAN^R	CAATGGCAGTGGTACAGGTTC	GCTCTTCGTCCAGATCATCCT	CGAAACCAGGCATCATCTTTG	GTCCGGTACCATAAAATCAACG	IDT DNA Inc.
Δ*lgoT*::KAN^R	GAAATTCCCGCTTACCTATGCTC	GCTCTTCGTCCAGATCATCCT	CGTGCCACCAGATGGATTAG	GATGTTCCGGCAAAGTGAATAC	IDT DNA Inc.
Δ*yghC*::KAN^R	CAGCTGCGGGATAACAAAATG	GCTCTTCGTCCAGATCATCCT	GTCATTCTTAGCCAGCAAATGG	GCACCAATAATTTGCCCATC	IDT DNA Inc.

Δ*tolC*::KAN^R	GTGAATTT CAGCGAC GTTTGAC	GCTCTTCGTC CAGATCATC CT	CCCATTCT TATCGGCC TGAG	GATGGGCA CTTTCCAGT TGC	IDT DNA Inc.
Δ*rnd*::KAN^R	GCTCACG GGATACA AAGTACC G	GCTCTTCGTC CAGATCATC CT	GCTGGCTT CTTTGTGT GAAGC	GTGTAATGC TTCCGCCAT CAG	IDT DNA Inc.
Δ*dld*::KAN^R	GCGAAAA TGGTGCA AAAACC	GCTCTTCGTC CAGATCATC CT	GAATGAA CTTGCTCG TCTGGTG	CAGTAAAAT GCGGACGG AAG	IDT DNA Inc.
Δ*acrB*::KAN^R	CAGGCTAT TGGCGAT AAGTGG	GCTCTTCGTC CAGATCATC CT	GGCGCAA TATCCTAC GATTG	CAATGGTCG GAATCAAC GTC	IDT DNA Inc.
Δ*ybcL*::KAN^R	GTGGCAA ATAATTGG GTTGG	GCTCTTCGTC CAGATCATC CT	GCTGCAG CATTTCAG GTCAC	GGTGTTATC TCAGCGGTT GC	IDT DNA Inc.
Δ*uidB*::KAN^R	GGTGAAC AGGTATG GAATTTCG	GCTCTTCGTC CAGATCATC CT	GTTACTGG TGCGGGT ATTCG	GATTGCCTG ACCGCATTT AC	IDT DNA Inc.
Δ*dsdC*::KAN^R	GGCTGAC AAACGAT AAAATTTG C	GCTCTTCGTC CAGATCATC CT	CCCTTCGT GAAATAA GAAATCG	GCTTCAATT TTTGGCCAC TG	IDT DNA Inc.
Δ*narJ*::KAN^R	CCTTCCCG GA GAAAAAT GGC	GCTCTTCGTC CAGATCATC CT	CTCGTGAT TGTATCGC GTCTC	CTGTCCTCC GGTGGTGAT ATTC	IDT DNA Inc.
Δ*yoeH*::KAN^R	GCGGTTGC AGTAGAA ACTATCC	GCTCTTCGTC CAGATCATC CT	GTCCGTGG AAAACGG AGTTG	CCGCATATA CGGTCAATT TCAG	IDT DNA Inc.

Δ*recF*::KAN^R	GGAAGAG ATCCTCGA CGTTACC	GCTCTTCGTC CAGATCATC CT	GCTTGTTG ATCCGCG ATTTC	CCCTTTTCC ACGGTAAA CATC	IDT DNA Inc.
Δ*rhsC*::KAN^R	TTGTATCA GGAAGAT GTTCAGA GGA	GCTCTTCGTC CAGATCATC CT	GCAGTAT GGCGGTA GCATTG	GTGGTGATA ACGCTGTGT GC	IDT DNA Inc.
Δ*dinB*::KAN^R	GACCAAA AGTGCGTC CGATAC	GCTCTTCGTC CAGATCATC CT	CCTGCGCG ATATCCCT ATTG	CCAGCACCA GTTGTCTTT CC	IDT DNA Inc.
Δ*flgN*::KAN^R	CCAGCAC CAGTGTG ACGTTAA G	GCTCTTCGTC CAGATCATC CT	CGTCTTGC AGAGATC CTCGAC	CGATGGGTT GTTGAGGTC TG	IDT DNA Inc.
Δ*hybE*::KAN^R	GCGAGTTT CCGAAAA AGCTG	GCTCTTCGTC CAGATCATC CT	CAAGTAC AGGCAGC GTTTGAAG	GTGGTACAT CCGGATTCG TG	IDT DNA Inc.
Δ*yjeP*::KAN^R	CTGGTTGA GCAACTG GAAAGC	GCTCTTCGTC CAGATCATC CT	CACTTTTC TGATGGCC TGGTG	GTTGTGGTT TCGGCATTT CC	IDT DNA Inc.
Δ*yghD*::KAN^R	GACATCG AAATGCG TGGTTTTA C	GCTCTTCGTC CAGATCATC CT	AGTTAAG CCGTGGC GAACAC	CATTCACCA TCCCAGGCT TC	IDT DNA Inc.
Δ*yfjR*::KAN^R	CATCGCTA TGTGCTCG TGTG	GCTCTTCGTC CAGATCATC CT	CTGAGTTT GGCGTGTC AGTG	GCCGGAAC AAACGTAA GCAG	IDT DNA Inc.
Δ*pgl*::KAN^R	AGGTGCC GTCTAAGT CGAGAG	GCTCTTCGTC CAGATCATC CT	CCCTGAG AGCCAGC AAATTC	CATAGCGGC CTTTTTCAT GC	IDT DNA Inc.

Δ*putA*::KAN^R	CTGCCGCAGAGAAAAAGTCTG	GCTCTTCGTCCAGATCATCCT	CGACGCGTGAGCGTATTAAG	CCAGCAGGGTGAGTGATTTC	IDT DNA Inc.
Δ*amiB*::KAN^R	GTGGCCACAACAAGGTACAGG	GCTCTTCGTCCAGATCATCCT	CGCGACGCTCTCTGATATTC	GACTGACTGTTCGCCAGCAC	IDT DNA Inc.
Δ*rlmB*::KAN^R	GGGTTAGATTGCTGACAAAATGC	GCTCTTCGTCCAGATCATCCT	GCGAAATGATTTACGGCATC	GCACCGCTTCAAATAAGCAA	IDT DNA Inc.
Δ*yodB*::KAN^R	CCATACACAATACGGCCAACAG	GCTCTTCGTCCAGATCATCCT	CCGTGGCTGGTTTCCTAAAG	GGCGAGGCATCATTCGTAAC	IDT DNA Inc.
Δ*kdpA*::KAN^R	CAGCCAGAATTCTACCCTTCC	GCTCTTCGTCCAGATCATCCT	CTGCGCAAGGGTTCTTACTG	GACCGCAAACAGGCTACTGAC	IDT DNA Inc.
Δ*appC*::KAN^R	CGGCTTAGCGAGGTATGTCAG	GCTCTTCGTCCAGATCATCCT	GGTCACCGGCAAAACAATCTAC	GCGGAAACTCCAGAATACCG	IDT DNA Inc.
Δ*arpA*::KAN^R	CAAAATCGCAAGATCTCATTCC	GCTCTTCGTCCAGATCATCCT	CAGCCCAGACAAATGAACACC	GGAGGGGTTATTTCATCCACAG	IDT DNA Inc.
Δ*pbpC*::KAN^R	AGCACATTGAGTTCCGTGAC	GCTCTTCGTCCAGATCATCCT	GCGCCCTTTCTCCTTTTTCT	CGGTTTGAGCACTGATCCTG	IDT DNA Inc.
Δ*rpmI*::KAN^R	GAAAGACGATTTGCAAGAACTGG	GCTCTTCGTCCAGATCATCCT	GCTGCTAAGCGCTTCAAAAA	CAGGCACGCGATTACCAG	IDT DNA Inc.

Δ*prpC*::KAN^R	GACAACC GATGCCTG ATGC	GCTCTTCGTC CAGATCATC CT	GACACAA CGATCCTG CAAAAC	GATTGGGG AACATCTTT TTGC	IDT DNA Inc.
Δ*yraP*::KAN^R	CGCGTGAT ATGACCAT TGTG	GCTCTTCGTC CAGATCATC CT	GCCAATC GCAGTCCT TATTTC	GCCGTAGTT ACCCGCTTC AC	IDT DNA Inc.
Δ*qseC*::KAN^R	CCCTGCTG GAATTACT GATGC	GCTCTTCGTC CAGATCATC CT	GGCTGCTT TCCAGCTT TGTC	GTTTGATGC TGTGGGCAT TG	IDT DNA Inc.
Δ*yhaL*::KAN^R	GGAAATTT CATGCGCT TACG	GCTCTTCGTC CAGATCATC CT	GTGGTGG CGAAAGA ACCTG	GGTAGCTTC ATCCTCGGC TAAAC	IDT DNA Inc.
Δ*iaaA*::KAN^R	CCACAGA CTGTATGC CACCTC	GCTCTTCGTC CAGATCATC CT	CGCAGAT GAGTCTGC AACAG	CACGGTAG ATACCGGTG GTTG	IDT DNA Inc.
Δ*malS*::KAN^R	GATCGCTC AC CCTTGCTT CTC	GCTCTTCGTC CAGATCATC CT	CGCCTGTT TTCTGACA CTCC	GAAAGATA TAACGCGCC AAACTG	IDT DNA Inc.
Δ*aroE*::KAN^R	CCAGTGCC AACTTGA GTGGA	GCTCTTCGTC CAGATCATC CT	GGTAATCC GATAGCC CACAGC	GAACACCGT GCCAGAGA AGA	IDT DNA Inc.
Δ*ugpQ*::KAN^R	GATGGTG ATGGACA CGCTGG	GCTCTTCGTC CAGATCATC CT	GGGCAAA ATACGGTC ATAAGAT G	CCGTAAAGT TCGGACCAA TCA	IDT DNA Inc.
Δ*rhsB*::KAN^R	GAACGAT CAACAAA GGGCCA	GCTCTTCGTC CAGATCATC CT	GCAGTAT GGCGGTA GCATTG	CTGTCGTAA CGGTAACG GATTTC	IDT DNA Inc.

Δ*yidF*::KAN^R	CGCTGATC CTCTGGCA CTAC	GCTCTTCGTC CAGATCATC CT	ACAGGAA GTCAGGTC ATTGATGC	CATCAGCTC CATGGGAG AAC	IDT DNA Inc.
Δ*phnG*::KAN^R	TGAAATCC CGAATAT GTCGC	GCTCTTCGTC CAGATCATC CT	CCTGAAC GCGCTAA ACATCA	CTCCGCGAA CCATCGTAA AG	IDT DNA Inc.
Δ*dgcQ*::KAN^R	TTAACATC AG GCCGGAT CAC	GCTCTTCGTC CAGATCATC CT	CACGAGA CAAAAAT GGAAAAC C	GCACACCGT CGAAATGGT C	IDT DNA Inc.
Δ*ygfF*::KAN^R	GAAAGGG CGA GGGGGAA AAG	GCTCTTCGTC CAGATCATC CT	GCATTACT GTTGGCGC AAG	CCGTGACGT AAGAGGCTT TATC	IDT DNA Inc.
Δ*adk*::KAN^R	CAGCTGCT GGTTTCCT GATGTA	GCTCTTCGTC CAGATCATC CT	GGGACTC AGGCTCA GTTCATC	CGTCAACTT TCGCGTATT TGG	IDT DNA Inc.
Δ*gpmM*::KAN^R	CAGCGCG TTAACTGG AATG	GCTCTTCGTC CAGATCATC CT	GCTATCGC GAAGAAC AGCAG	GGTTTCGGA AATACGCA ACTG	IDT DNA Inc.
Δ*gltA*::KAN^R	GATTCGCC ATTTATTC GTCATC	GCTCTTCGTC CAGATCATC CT	CACCCTCA ACGGGGA TACAG	CAGCTCTTT CAGCACTTC ATGG	IDT DNA Inc.
Δ*fau*::KAN^R	CGTCCGA GAAGCCTT AAAACTG	GCTCTTCGTC CAGATCATC CT	GTTAACGC CGGAACA ACAGC	GGTGTAACC ACCGCAGG AAG	IDT DNA Inc.
Δ*nuoA*::KAN^R	GTTGACA AAAGGTT ATAGAAA GGAGT	GCTCTTCGTC CAGATCATC CT	GTCAACAT CCACTGA AGTCATCG	GATACTGTT CGTTTCCGG GTTC	IDT DNA Inc.

Δ*uvrD*::KAN^R	CAGGCTTG TTGGATCA GACC	GCTCTTCGTC CAGATCATC CT	GTAAGAC GCGCGTA CTGGTG	GTTGCTGCG GTAGAGAA TGG	IDT DNA Inc.

Table S5: Chemicals used in this study.

**Chemicals**	**Purity (%)**	**Source**	**Catalog number**
Ofloxacin	98	Fisher Scientific	AC455670050
Kanamycin	>95	Fisher Scientific	AC450811000
Isopropyl ß-D-1-thiogalactopyranoside	>99	Fisher Scientific	BP1755-10
